# The Swedish Malaise Trap Project: A 15 Year Retrospective on a Countrywide Insect Inventory

**DOI:** 10.3897/BDJ.8.e47255

**Published:** 2020-01-21

**Authors:** Dave Karlsson, Emily Hartop, Mattias Forshage, Mathias Jaschhof, Fredrik Ronquist

**Affiliations:** 1 Station Linné, Färjestaden, Sweden Station Linné Färjestaden Sweden; 2 Stockholms Universitet, Stockholm, Sweden Stockholms Universitet Stockholm Sweden; 3 Swedish Museum of Natural History, Stockholm, Sweden Swedish Museum of Natural History Stockholm Sweden

**Keywords:** All-taxa biodiversity inventory (ATBI), biota, diversity, entomology, inventory, insects, Malaise Trap, community science, citizen science

## Abstract

The Swedish Malaise Trap Project (SMTP) is one of the most ambitious insect inventories ever attempted. The project was designed to target poorly known insect groups across a diverse range of habitats in Sweden. The field campaign involved the deployment of 73 Malaise traps at 55 localities across the country for three years (2003-2006). Over the past 15 years, the collected material has been hand sorted by trained technicians into over 300 taxonomic fractions suitable for expert attention. The resulting collection is a tremendous asset for entomologists around the world, especially as we now face a desperate need for baseline data to evaluate phenomena like insect decline and climate change. Here, we describe the history, organisation, methodology and logistics of the SMTP, focusing on the rationale for the decisions taken and the lessons learned along the way. The SMTP represents one of the early instances of community science applied to large-scale inventory work, with a heavy reliance on volunteers in both the field and the laboratory. We give estimates of both staff effort and volunteer effort involved. The project has been funded by the Swedish Taxonomy Initiative; in total, the inventory has cost less than 30 million SEK (approximately 3.1 million USD). Based on a subset of the samples, we characterise the size and taxonomic composition of the SMTP material. Several different extrapolation methods suggest that the material comprises around 20 million specimens in total. The material is dominated by Diptera (75% of the specimens) and Hymenoptera (15% of specimens). Amongst the Diptera, the dominant groups are Chironomidae (37% of specimens), Sciaridae (15%), Phoridae (13%), Cecidomyiidae (9.5%) and Mycetophilidae (9.4%). Within Hymenoptera, the major groups are Ichneumonidae (44% of specimens), Diaprioidea (19%), Braconidae (9.6%), Platygastroidea (8.5%) and Chalcidoidea (7.9%). The taxonomic composition varies with latitude and season. Several Diptera and Hymenoptera groups are more common in non-summer samples (collected from September to April) and in the North, while others show the opposite pattern. About 1% of the total material has been processed and identified by experts so far. This material represents over 4,000 species. One third of these had not been recorded from Sweden before and almost 700 of them are new to science. These results reveal the large amounts of taxonomic work still needed on Palaearctic insect faunas. Based on the SMTP experiences, we discuss aspects of planning and conducting future large-scale insect inventory projects using mainly traditional approaches in relation to more recent approaches that rely on molecular techniques.

## Introduction

When the great Swedish naturalist Carl Linnaeus set out to describe and classify life on earth, he estimated there would be fewer than 30,000 species to name and characterise (Linnaeus 1735). Despite successive editions of his great taxonomic opus *Systema Naturae*, he would ultimately cover only a small fraction of the world’s biodiversity in his lifetime (Linnaeus 1758). Two-hundred and fifty years later, current estimates suggest that the number of species, including unicellular organisms, on earth may be anywhere from 8.7 million to one trillion, with just 1.2 million currently described (Locey and Lennon 2016, Mora et al. 2011).

With so much work before us, how do we design inventories to efficiently collect, sort, identify and store the world’s abundant biodiversity? The sheer number of species on earth means that few modern inventories can be either taxonomically or geographically comprehensive. Even in our modern era of bioinformatics and next generation sequencing technologies, cataloguing the world’s biota remains a huge scientific challenge; we are not even close to completing the task. Current inventory projects are, by necessity, limited by taxonomic groups, regions or (most often) both. The Planetary Biodiversity Inventories Program launched by the US National Science Foundation in 2003 supported a taxonomic approach to inventories by funding international teams charting the world flora and fauna of select taxonomic groups. An alternative approach is to focus on describing the biota of a circumscribed geographic area, a concept initially conceived by Daniel Janzen for a comprehensive study of the Guanacaste Conservation Area in Costa Rica (Janzen and Hallwachs 1994). This approach is now widely known as an All Taxa Biodiversity Inventory (ATBI) and numerous ATBIs are currently underway in different parts of the world. One of the oldest and most successful is the ATBI in the Great Smoky Mountains National Park (GSMNP) in the south-eastern United States (https://dlia.org/smokies-species-tally). This inventory was initiated in 1998 by the GSMNP and is now supported jointly by the park and a non-profit organisation, Discover Life in America. The GSMNP ATBI is exceptional in its longevity, having established a non-profit organisation specifically to secure its efforts. The long running success of the project has inspired other ATBIs throughout the US National Park system. Unfortunately, most ATBIs are not as well coordinated or funded, resulting in slow progress and persistent doubts about whether the ultimate objective is achievable. An interesting recent effort, which combined the ATBI approach with a taxonomic focus, attempted to chart all the species of Diptera – one of the megadiverse orders of insects – on a 4-hectare plot of tropical cloudforest at 1600 metres at Zurqui de Moravia, Costa Rica. This project, the Zurqui All Diptera Biodiversity Inventory (ZADBI), revealed an astounding diversity of morphospecies and abundance of specimens (Brown et al. 2018, Borkent and Brown 2015, Borkent et al. 2018).

Tropical regions, such as those studied by Janzen or the ZADBI project, offer vast, unexplored biological richness and myriad small and diverse creatures in abundance; they are easy targets with plenty of low hanging fruit for the taxonomist or intrepid explorer. In contrast, much of the insect richness of the Palaearctic region is well-documented (and has been for centuries). What is left are many of the least charismatic groups; often some of the smallest in size, but the most diverse, ubiquitous and taxonomically challenging. Properly focusing on these groups remains the major challenge of actually completing any ATBI. Arguably the most persistent, ambitious and well-funded ATBI ever attempted is the Swedish Taxonomy Initiative/Svenska artprojektet (STI), a funding source that devotes the majority of its resources to supporting work on such understudied taxa.

The STI was commissioned by the Swedish Parliament and has been funded by the Swedish government continuously since its inception in 2002, coordinated by the Swedish Species Information Centre/Artdatabanken (SSIC) at the Swedish University of Agricultural Sciences (Gärdenfors et al. 2003, Miller 2005). Its original goal was to chart, document and provide identification tools for all macroscopic flora and fauna of Sweden within 20 years (Ronquist and Gärdenfors 2003). With its Linnaean tradition and public appreciation of nature, Sweden is a natural home for an ambitious national ATBI (Ronquist and Gärdenfors 2003). Sweden is, admittedly, a wealthy nation with relatively modest biodiversity, but nevertheless, it is estimated to have a respectable 60,000 native species of multicellular organisms, many of which are poorly known or undocumented – thus, a comprehensive ATBI remains a challenge (Ronquist et al. 2019).

It was clear from the outset that the insect fauna would require special attention even though Sweden has longstanding entomological and taxonomic traditions. One of the first attempts to document the Swedish fauna is Linnaeus’s *Fauna suecica*. The first edition appeared in 1746, but a more in-depth treatment with modern binomial names was not published until the second edition in 1761 (Linnaeus 1746,Linnaeus 1761). Linnaeus listed almost 1,500 insect species for Sweden. Following this, entomologists trended towards treating separate insect orders (or parts thereof) in monographs and/or dissertation series. Accounts of the country’s fauna listed species and often small numbers of geographical records with largely anecdotal focus, including scattered taxonomic revisions with basically no conceptualisation of what we would now consider ecological or biogeographical aspects. In this manner, amongst the famous ”classical authors”, Thunberg, Dalman, Fallén and Zetterstedt worked with most insect orders (and the latter two especially with Diptera), whereas others focused principally on Coleoptera (Paykull, Gyllenhal, Schönherr) or Hymenoptera (Dahlbom, Holmgren) or smaller orders, but notably few on Lepidoptera (the first proper specialist was Wallengren).

It can be argued that the first ”national entomologist” who travelled and collected over as much as possible of the country, as well as one of the relatively few who dealt with most or all insect orders, was C H Boheman, the director of entomology at the new national natural history museum in Stockholm from 1841. In time and taxonomic scope, he partly overlapped with the much younger, Skåne-focused but taxonomically equally broad, C G Thomson, who was responsible for the first summarising account of Sweden’s insect fauna after Linnaeus (Thomson 1885), estimating ca. 11,000 species for Scandinavia. Around this time, the first provincial catalogues of ”Macrolepidoptera” and Coleoptera were edited, based on national museum collections (Lampa 1885, Grill 1896). Soon, the continuing series of traditional monographs were replaced by far more synthetic, heavily edited and popular accounts of principal insect groups in the series ”Svensk insektfauna” (starting in 1901).

During the early decades of the 20th century, coleopterists and lepidopterists struggled to fill the blank areas on the distribution map systematically. Now, species distributions were not simply wherever each species happened to have been caught, but were logical patterns based on habitats, species assemblages (”biocoenoses”), reflecting, in part, geological and meteorological conditions. Around this time, two of the era's most well-known and prolific entomologists compiled the vast book *Svenska insekter* (Tullgren and Wahlgren 1922). This remains the last comprehensive synthesis of the Swedish insect fauna, documenting the presence of about 15,400 insect species for the nation (Tullgren and Wahlgren 1922).

In the last century, Coleoptera and Lepidoptera have been scrutinised and catalogued at regular intervals, whereas the hyper-diverse Diptera and Hymenoptera have yet to be nationally catalogued. Mid-century, an admirable insect handbook series was begun but aborted halfway (Landin 1971). There was thus no reasonably current overview of the Swedish fauna available when the STI was launched in 2002.

The focus of this paper is the ambitious countrywide insect inventory funded by the STI, the Swedish Malaise Trap Project (SMTP). The project was designed to target poorly known insect groups across a diverse range of habitats in Sweden; the field campaign involved the deployment of 73 Malaise traps at 55 localities across the country for three years (2003-2006). Continued funding of the SMTP effort by the STI over the past 15 years has allowed the entire collected material — 1919 samples comprising an estimated 20 million specimens — to be sorted by trained technicians into more than 300 taxonomic fractions suitable for expert attention. The processed SMTP collection represents a tremendous resource for entomologists around the world. Thus far, about 1% of the material has been identified to species by experts, especially targeting the poorly known and species-rich insect groups. This work has revealed a surprising amount of previously unknown insect diversity in Sweden.

In this paper, we describe the history of this entomological megaproject to provide a record for entomohistorians of the future. Additionally, we provide a resource for researchers analysing the SMTP material by providing relevant background information on the project. Finally, by describing the logistics and lessons learned in organising the SMTP project in some detail, we hope to facilitate and provide information about the planning of future inventory projects in other parts of the world.

We hope this retrospective comes at a time when the impetus to catalogue biodiversity is growing and that others can glean both inspiration and information from our experiences. As we lose species at an increasingly alarming rate due to human activity, there must be a push to tackle the hyperdiverse, but poorly known, taxa that have so long been neglected. Recent reports suggesting a shocking decline in insects populations have revealed that there is a lack of solid baseline data available to explore such phenomena across the globe (Hallmann et al. 2017, Lister and Garcia 2018, Sánchez-Bayo and Wyckhuys 2019). The SMTP is sitting on a potentially pivotal source of data in the form of a massive, sorted insect collection that represents three years of data from over a decade ago. We hope that we can, not only inspire others to utilise this resource, but to organise inventories across the globe now to secure similar baseline data for the future.

## Material and methods

### Background

The first call for proposals from the STI was announced in early 2002 and Fredrik Ronquist (then at Uppsala University) and Thomas Pape (then at the Swedish Museum of Natural History) submitted a proposal for an inventory of Swedish Hymenoptera and Diptera (Sundin and Gärdenfors 2014). The original plan involved the deployment of 50 Malaise traps across Sweden for three years, complemented by yellow pan traps at selected sites. Specific taxa of parasitic Hymenoptera and the most diverse and poorly known families of Diptera were to be culled from the material, sorted to suitable taxonomic fractions and then made available for taxonomic research with priority given to projects funded by STI. The project would thus cover large holes in two of the most diverse groups of insects that were poorly represented in existing museum collections, a huge contribution to the STI’s pursuit of an ATBI of Sweden.

The project was funded in late 2002 and, in early 2003, Paul Hanson of the University of Costa Rica was invited to the Swedish Museum of Natural History to share his experiences from a similar inventory project in Costa Rica (Hansson 1999). Project plans were finalised in the following months and the field campaign launched in the summer of 2003. The following year, Dave Karlsson took over as project manager and the project headquarters were moved from the Swedish Museum of Natural History to the Ecological Field Station of Uppsala University (run by an independent non-profit organisation since 2008 as “Station Linné”) on the Baltic island of Öland. On Karlsson’s initiative, the scope of the project was widened to include all insects and the project name was changed to the “Swedish Malaise Trap Project” (SMTP) in 2006. The project is still run by Station Linné, while the collections generated from the project belong to and are managed in collaboration with the Swedish Museum of Natural History (NRM).

### Trapping Method

The initial SMTP operating budget supported just a single employee in 2003, Johan Liljeblad (SMTP project manager 2002-2004). Therefore, it was necessary to rely on volunteers to manage field sites and it was decided to use Malaise traps as the sole collection method because of their efficiency in collecting target groups (Diptera and Hymenoptera) and their ease of operation.

The Malaise trap, invented by the Swedish entomologist René Malaise (Malaise 1937, Sjöberg 2015, Vårdal and Taeger 2011), is a simple tent-like structure that traps insects and other small organisms by passively obstructing their flight or drift paths and then relying on their tendency to move upwards or towards light to make sure they end up in the collecting jar. In addition to flying insects, Malaise traps also catch a significant number of flightless organisms that climb up the trap and into the collecting head, some after first being carried into the trap passively by winds, but in many cases because of a tendency for insects to move up and down vegetation during the day. Phoretic forms that otherwise are not very mobile also end up in trap catches carried by their hosts. The Malaise trap is least effective in catching large, active insect flyers with good vision, such as dragonflies and butterflies, although these do end up in the catch in small numbers. A Malaise trap can be left without emptying for a week or longer, depending on the season and the emptying routine involves a quick shift of jars. Most other types of traps must be emptied more frequently, typically every two or three days and the emptying routines are more laborious. Malaise trap catches are also clean, facilitating sorting of the samples; samples generated by other trapping methods may contain various amounts of debris that slow processing.

**Figure 1.**
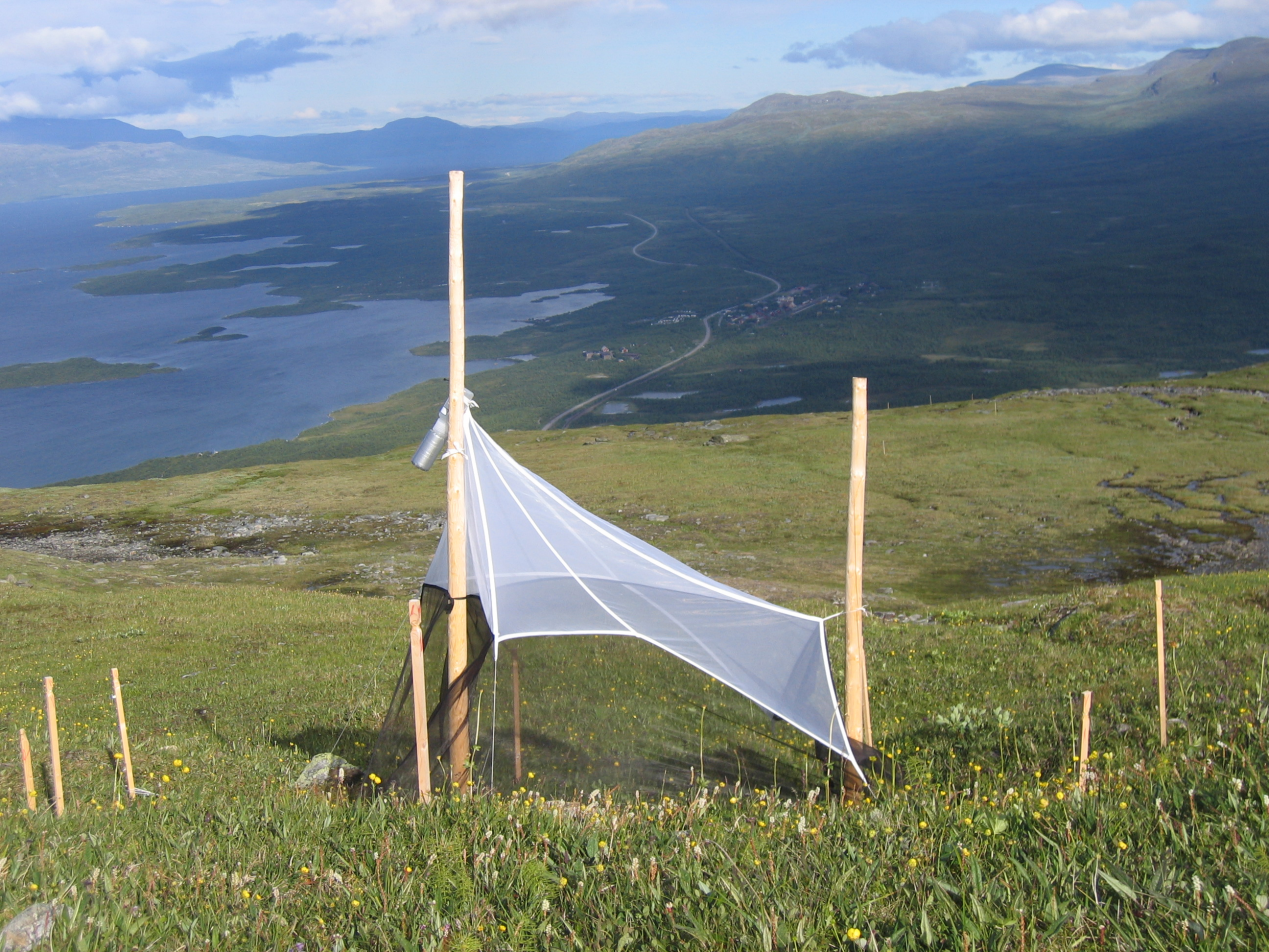
A Townes-style Malaise trap in position at Abisko, with surrounding fencing added to protect the trap from reindeer.

The design of a Malaise trap can vary broadly with respect to size, colour and shape, which are all known to affect the size and composition of the catch (van Achterberg 2009, Townes 1972, Roberts 1970). The fabric composition is of utmost importance; only very fine mesh (mesh size below 200 µm) or a fine tricot warp knit (such as that in the traps used by the SMTP) will effectively catch the tiniest insects, such as some very small microhymenopterans. For the SMTP, Townes-style Malaise traps with black walls and white roof (Fig. 1) made by Sante Traps (www.santetraps.com) were chosen. The light roof is meant to strengthen the phototactic response of trapped insects, funnelling them more rapidly into the collecting head (Townes 1972). The trap design is approximately 180 cm in both height and length. The collecting head consists of two interconnected 500 ml bottles made of semi-transparent polypropylene, with the upper bottle firmly attached to the trap and the lower bottle readily changeable. The lower bottle was wrapped in duct tape to protect the catch from excessive exposure to sunlight, as this is known to contribute to the degradation of DNA. Eighty percent ethanol was used in the collecting bottles. This was a compromise between the high concentrations preferred for DNA preservation and lower concentrations that leave specimens more malleable for morphological study and that evaporate at a lower rate under field conditions.

### Sites

During the initial planning, 140 localities were identified as prospective collecting sites. The list was partly inspired by a report from the Swedish Environmental Protection Agency listing habitat types that were particularly valuable with respect to Swedish biodiversity (Bernes 1994). These habitats included: (1) subarctic mixed pine-spruce-birch forest; (2) old pine forest; (3) nutrient-rich spruce swamp; (4) old boreal aspen forest; (5) boreo-nemoral pine-spruce-deciduous forest; (6) nemoral deciduous forest; and (7) nutrient-rich bog. Other types of habitats were identified as interesting because of their unusual fauna and flora or their peculiar geology. These included alvar, coastal rocks and meadows and fire-affected areas. It was also considered important to cover the range of climates and day-length regions in the country, which spans from 55 to 70 degrees North (almost 1600 km) and includes both coastal areas and a mountain range with peaks up to 2,111 metres above sea level. The final selection of sites was based on the availability of volunteers who could be relied on to operate the traps, while maintaining as much of the diversity and range across the country as possible from the original list. Permission for trapping sites was readily obtained from private landowners, nature reserves and national parks.

**Figure 2.**
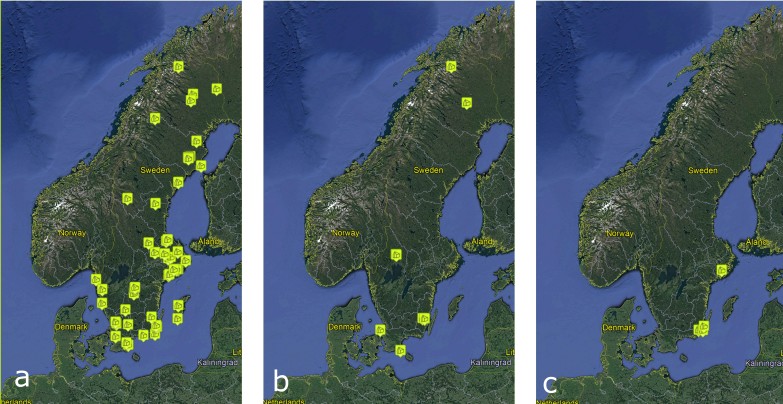
SMTP trapping sites. (a) The first 60 Malaise traps (Traps 1-61) erected in the summer of 2003. (b) Ten traps (Traps 1000-1008 and 1353) added in the winter of 2004/2005. (c) Three traps (Traps 2003, 2006 and 2046) added in 2006.

The first 60 Malaise traps (Traps 1-61, Trap 19 was never operational) were deployed at 44 localities throughout Sweden during the summer of 2003 (Fig. 2a). In 2004, several sites had to be terminated early, which left room for additional collecting efforts. Therefore, ten traps (Traps 1000-1008 and 1353) at six localities were added in the winter of 2004/2005 (Fig. 2b). The 70 traps that were still in operation in 2006 were then dismantled. The 2003–2006 campaign was later followed up by an additional, small campaign involving three traps (Traps 2003, 2006 and 2046) at two localities in 2006-2009 to complement the diversity of the original sample (Fig. 2c).

**Figure 3.**
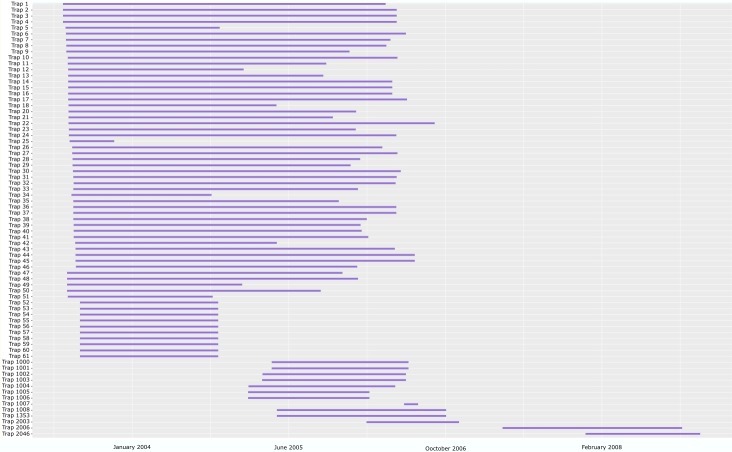
Timeline of SMTP trapping periods.

The core trapping period between 2003 and 2006 involved a total of 70 traps run for 53,062 days (1,745 trap months or 145 trap years) (Fig. 3). The two traps, run from 2006–2009, represent 1,206 trap days (40 trap months or 3.3 trap years). In total, the material generated from the two campaigns comprises 1,919 samples representing 149 trap years from 73 different sites. The main collecting campaign generated 1,868 (97.3 %) of samples.

### Setup and Maintenance

**Figure 4.**
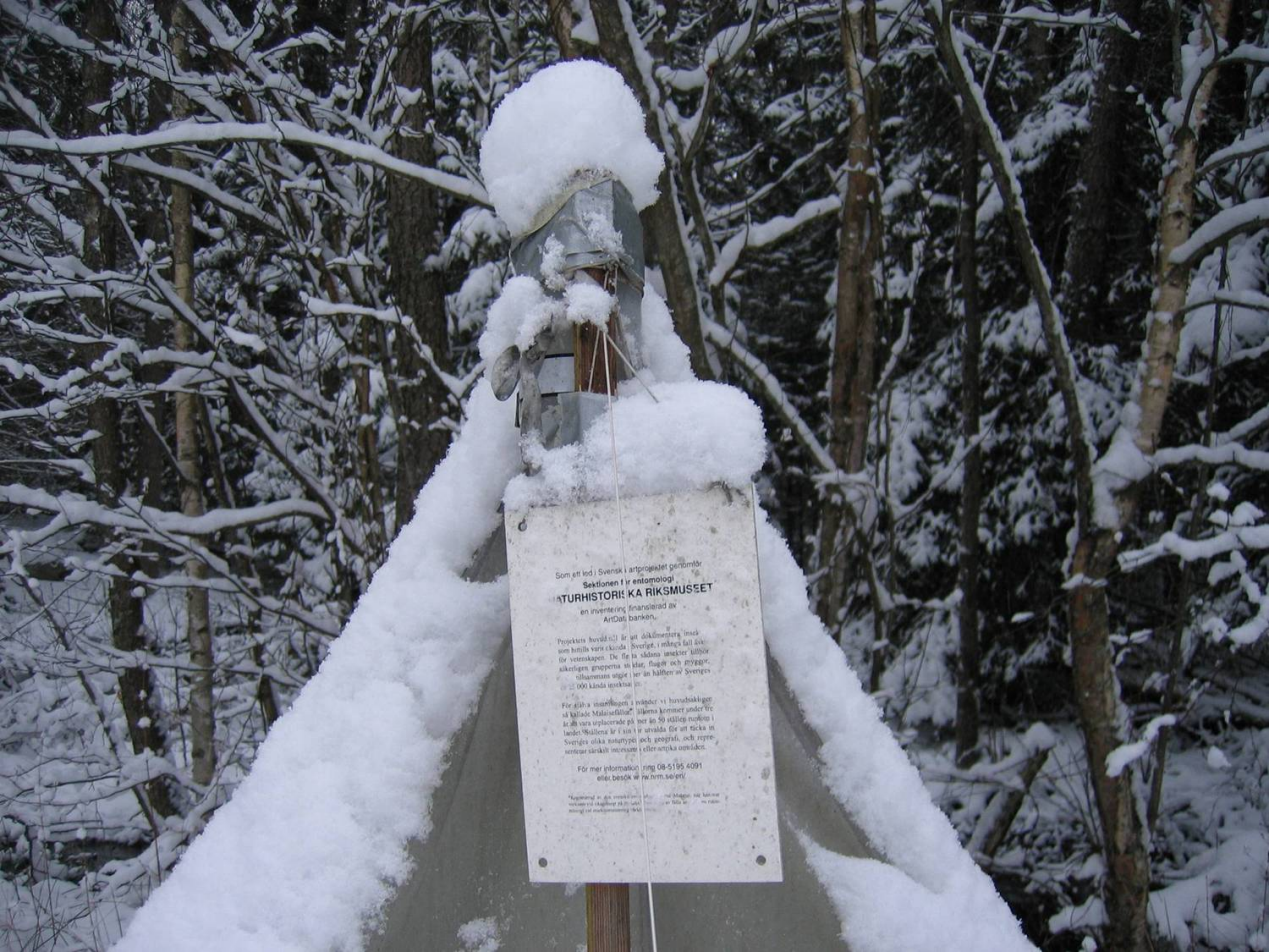
The sign placed on every SMTP Malaise trap in the field. The sign (in Swedish) describes the project, the trap and gives contact information.

Initial contact with volunteers, interested in being trap managers, was established by phone. After a trap manager was selected and had accepted, the trap was deployed by a visiting SMTP representative. During the visit, the trap manager received training in how to operate the trap and how to repair or replace it if needed. Every trap manager also received written instructions on how to operate the trap together with pre-printed labels for their trap(s), a graphite pencil suitable for adding notes to the collecting labels, a pack of resealable plastic ziplock bags for emptying the traps and a 5- or 10-litre container with 80% ethanol for refilling the collecting jar. To reduce the risk of traps being tampered with, a sign was attached to each trap providing information about the project and providing contact details (Fig. 4).

Trap managers were instructed to change the traps in approximate two-week intervals by emptying the collection jar into one of the provided ziplock bags following a standardised procedure that included washing the bottle with ethanol to remove any insects stuck inside. Collected samples were to be stored under as dark and cold conditions as possible until they were retrieved by SMTP staff. Finally, trap managers were instructed to check the traps as often as possible, to remove any spider webs that might be blocking the entry to the collecting head and to replace or repair any damage to the traps.

**Figure 5.**
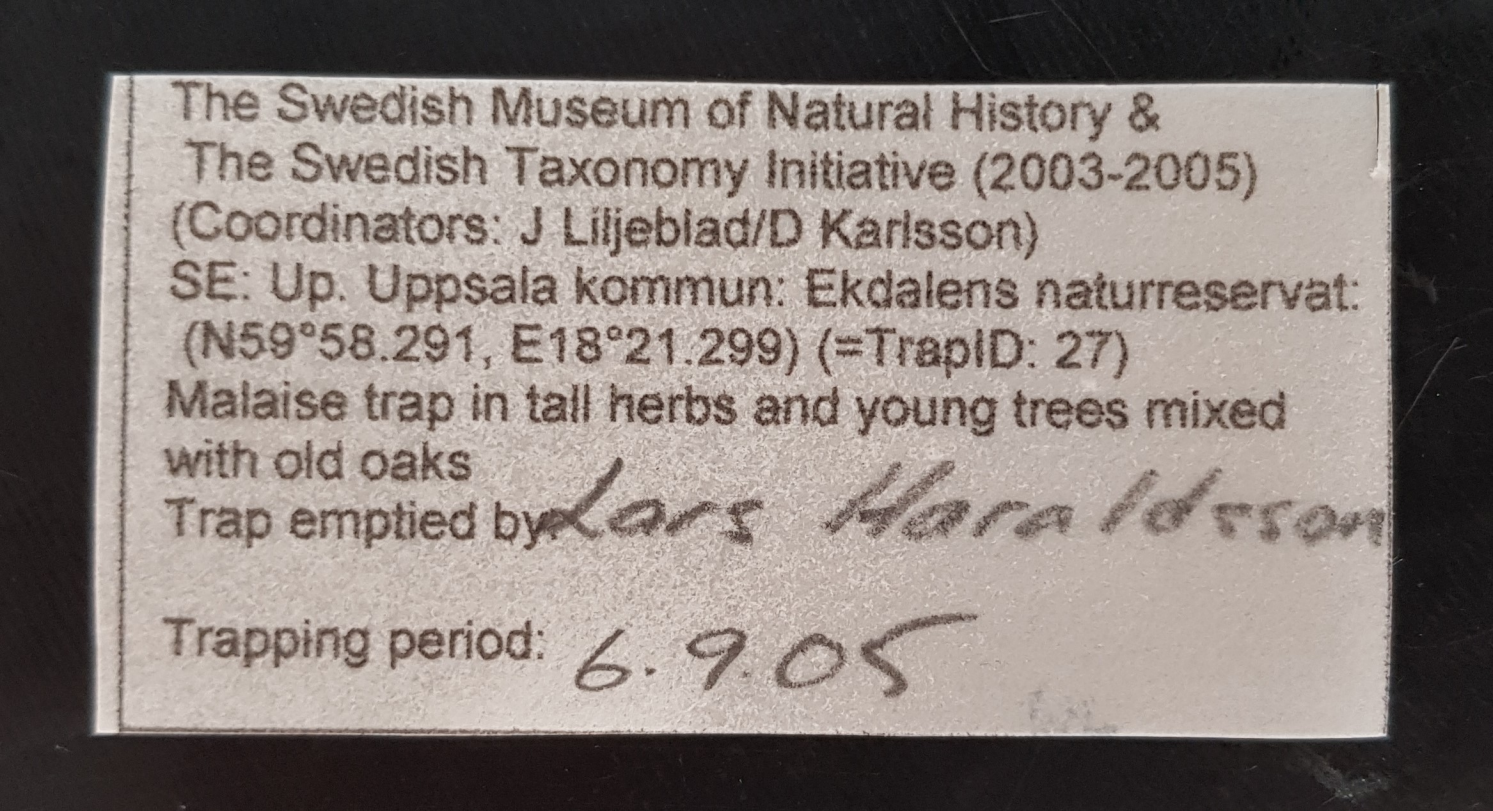
Sample label, as used in Malaise trap jars.

The preprinted sample labels measured approximately 60 by 30 mm, which was large enough to accommodate any additional, hand-written notes as needed. Labels were printed on high-quality, cotton-based 80-100 g Prime Archival paper (“Svenskt arkiv”), which is durable and suitable for both dry archiving (because it is acid free) and alcohol preservation (because of its high wet strength). Labels were printed by ink printer using pure black ink. The label data included all trap data and collection data specific to the sample (Fig. 5).

### Retrieval and Storage

Samples were retrieved from trap managers during scheduled collecting trips along three different routes: a southern route over 2,250 km covering the southernmost third of Sweden, a 3,000 km route through central Sweden and a 6,000 km route through the country's northern half. The southern route was serviced three times per year (spring, summer and autumn), the central route two times (early summer and late autumn) and the northern route once a year (late summer). Samples from the two isolated trap sites on the island of Gotland (Traps 28 and 29) and from the small follow-up collection campaign were handled separately.

**Figure 6.**
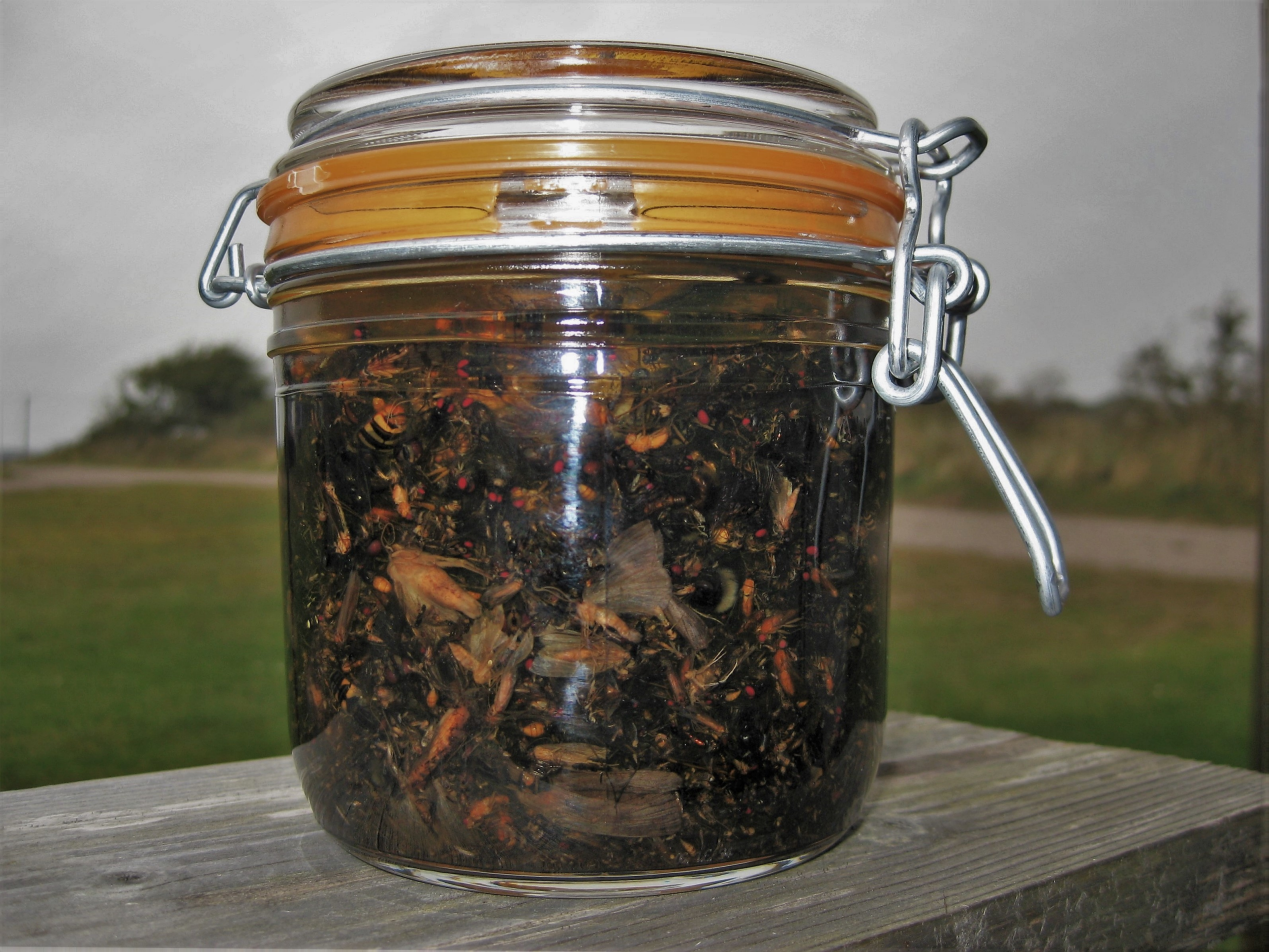
An unsorted Malaise trap sample.

Samples were retrieved from trap sites and brought to the Swedish Museum of Natural History in Stockholm, where they were transferred to standardised half-litre glass jars equipped with age-resistant silicone rubber seals (Fig. 6). The ziplock bags that initially held the samples were rinsed with 95% alcohol after the transfer to ensure that no specimen was lost. Larger samples were split into two or more jars for better preservation. Jars were filled no more than half-full of specimens for storage and were topped up with 95% ethanol. Each sample was then tagged with a unique collecting event number. Although ethanol concentrations were neither checked nor standardised, the use of 95% ethanol at this stage was designed to counteract any dilution of the original 80% ethanol that had occurred due to water leaking out from the insects in the sample.

The samples were initially stored in the wet-collection facility of the Swedish Museum of Natural History at 18°C, in darkness. The samples were subsequently transferred to Station Linné for sorting into taxonomic fractions. At Station Linné, the samples were stored in freezers at -18°C as space allowed. Alternatively, they were housed in refrigerators at +4°C or, when the capacity was exhausted, in darkness in storage rooms without climate control. Since 2016, all SMTP material in ethanol has been held in long term storage at -20°C.

### Sample Sorting

Although, from the outset, the number of specimens that would ultimately be sorted was unknown, it was clear that sorting the roughly 2,000 samples would require a significant time investment. This, consequently, meant that well-trained personnel who could expedite the process would be essential. It has been important over the years to continually optimise the workflow with respect to speed and accuracy of the identifications (and therefore the quality of the sorted material for subsequent work). The workflow has evolved significantly over more than a decade of sorting and the following describes the current protocol.

A raw sample is processed by transferring portions of the original sample into a sorting tray using a regular tablespoon and then sorting that portion into taxonomic units before refilling the sorting tray from the sample jar. In general, this begins by picking out large insects like bumblebees, butterflies/moths and beetles and carefully removing any small specimens that have become lodged in their legs, setae etc. (an exception would be phoretic insects, that may be left with their hosts). The sample then proceeds to smaller and smaller insects, with experienced technicians often creating small piles of the most abundant taxonomic fractions in their sorting trays and transferring them en masse to the appropriate sorting vials.

**Figure 7.**
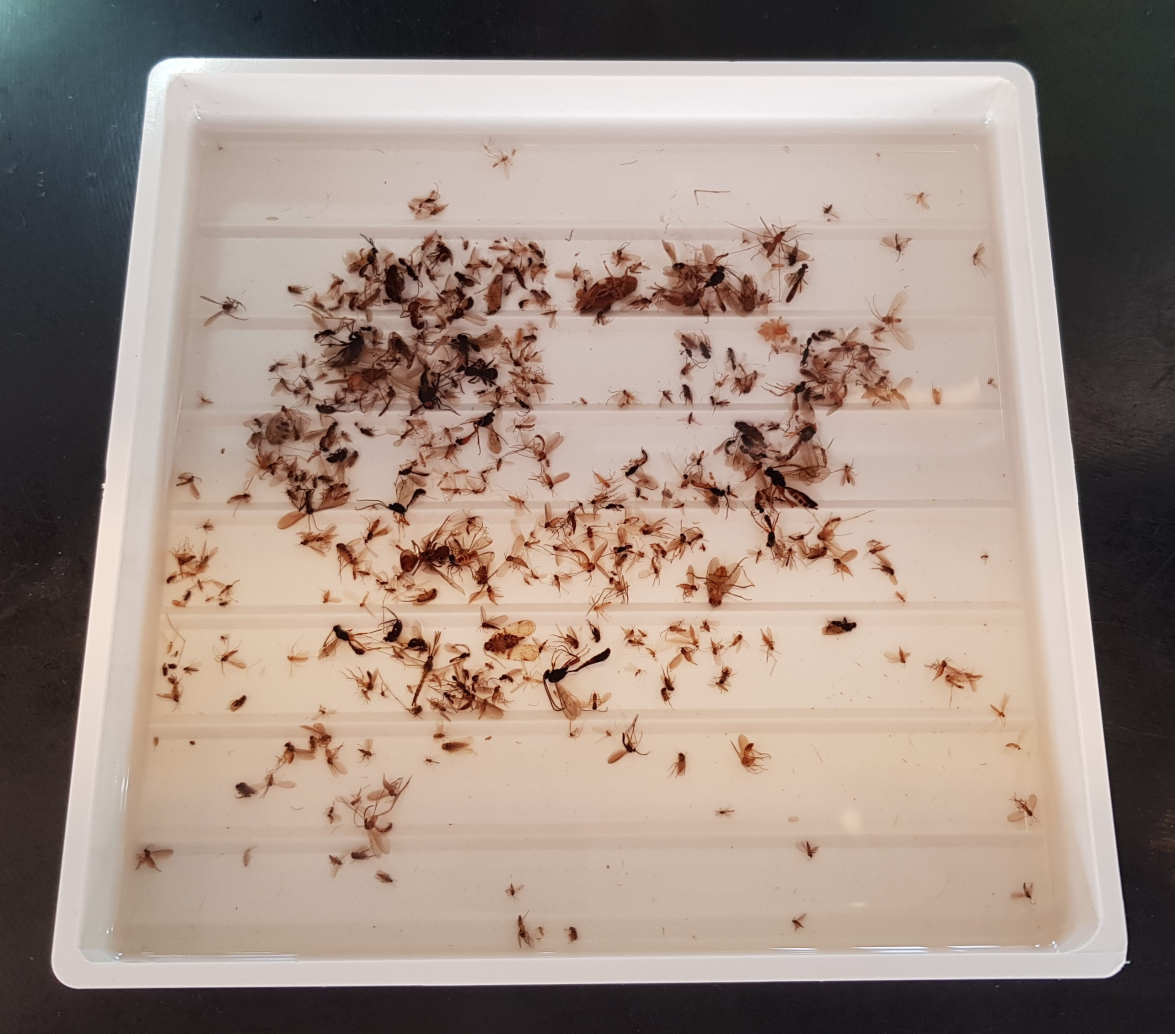
Sorting tray from Rose Entomology

Sorting is performed in 95% ethanol. To ensure that samples do not dry out in the trays, a minimum depth of 10-12 mm of 95% ethanol is used to cover the specimens during sorting. During breaks, a second sorting tray is inverted to cover the specimens to prevent evaporation of the alcohol. Most SMTP sorting is done using 11RST1 trays from Rose Entomology (now part of Bioquip, Rancho Dominguez, California, USA). These trays are specifically designed for sorting insect specimens in ethanol by means of a dissecting microscope. They are injection-molded from bright white ABS plastics, providing a high-contrast background and they have seven raised partitions creating lanes to organise sorting (Fig. 7). Most sorting is done with flexible, narrow-tipped stainless-steel forceps to minimise specimen damage. More rigid fine-tipped forceps are used for some larger insects and specimen cleaning, as needed. Malaise trap samples rarely contain much extraneous material that needs to be removed, but they may occasionally contain large numbers of lepidopteran scales that can obscure the view during sorting. These can rather effectively be removed by gently touching the liquid surface of the sorting tray with pieces of high-absorbency soft paper (such as lint-free paper towels).

**Figure 8.**
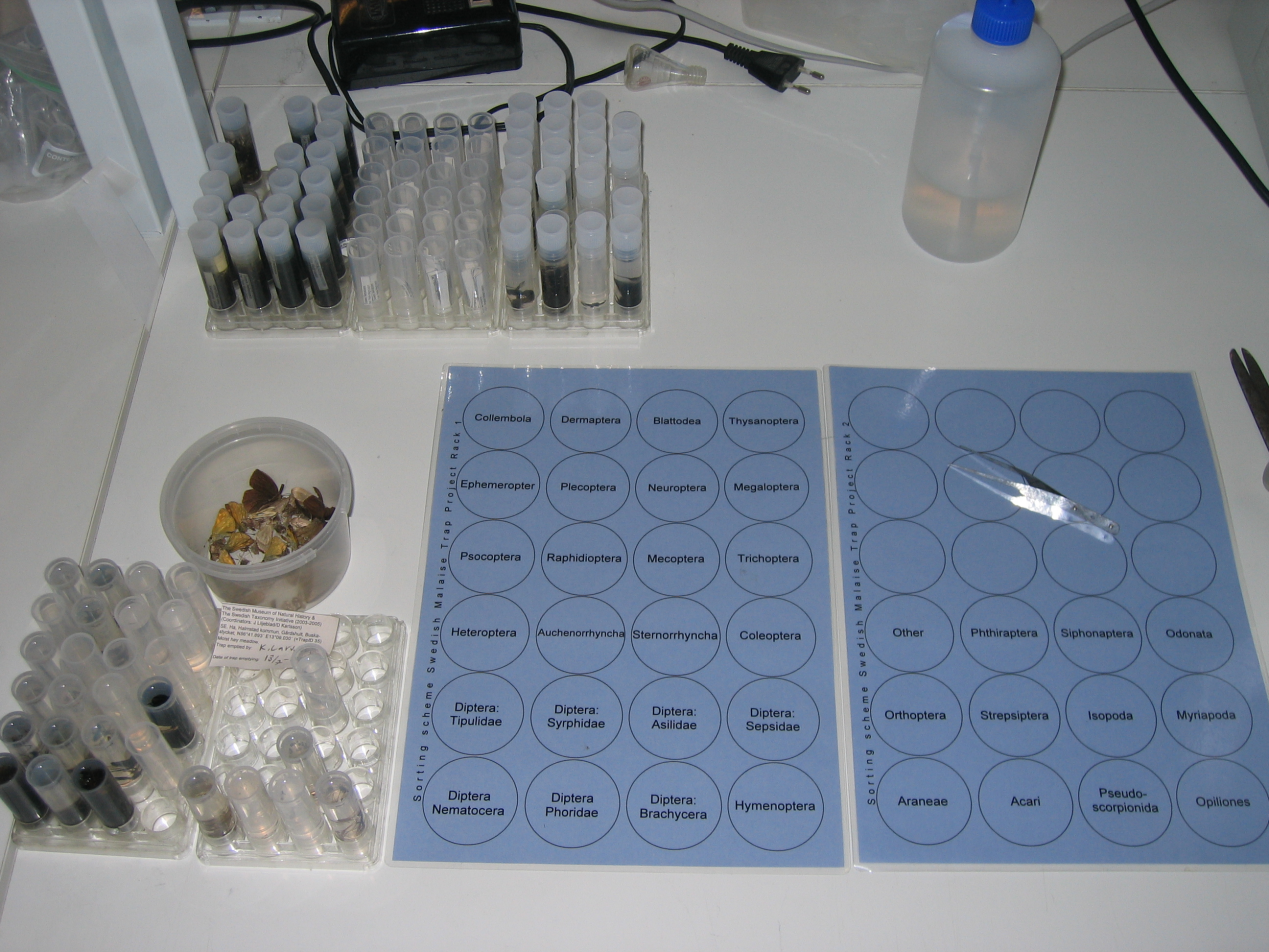
Workstation setup for SMTP technicians showing sorting guide.

Sorting is performed according to a tiered system. In the first tier, the sample is sorted into 35 taxonomic fractions generally corresponding to insect orders. Specimens are placed in 7 ml Sarstedt tubes of 95% ethanol as they are identified and these are organised in transparent polycarbonate racks placed over printed sorting schemes (Fig. 8). Lepidoptera specimens are placed on a paper towel at the sorting station and are allowed to dry before being moved into plastic containers with tightly fitting lids for storage. Of the 35 fractions from the first sorting tier, 32 are considered adequate for immediate further processing by taxonomic experts. The remaining three fractions – Hymenoptera, Diptera: “Nematocera” and Diptera: Brachycera – are hugely diverse clades or paraphyletic assemblages that require further sorting before the material is appealing to specialists. Therefore, these groups go through a second tier of sorting, to the superfamily or family level. In Hymenoptera, two megadiverse families, the Ichneumonidae and Braconidae, are further sorted to subfamily level. Finally, some subfamilies of the Ichneumonidae are sorted beyond this to tribe or genus. In total, SMTP samples are sorted into more than 300 taxonomic entities (http://www.stationlinne.se/en/research/the-swedish-malaise-trap-project-smtp/taxonomic-units-in-the-smtp/).

**Figure 9.**
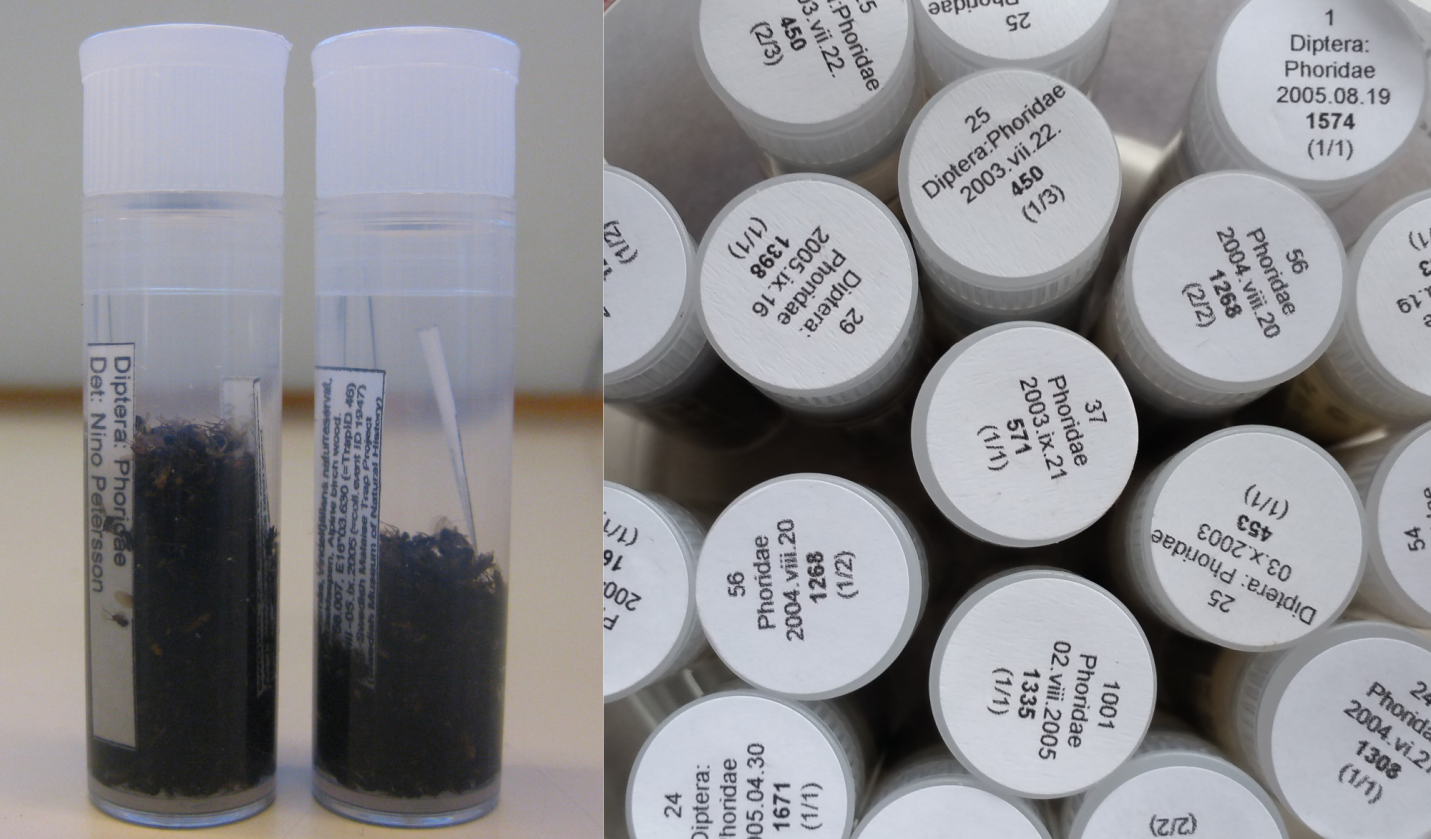
SMTP sorted fractions labelling (a) Interior labels with trapping information, sorter and taxon, (b) Exterior labels with summarised information for ease of location.

A vial is considered full when the insects occupy half to two thirds of its total volume. Two pre-printed labels, a collecting data label and a sorting data label, are then added (Fig. 9a). The collecting data label contains both trap and collecting event information in six lines. Its size is 25 x 10 mm and it uses the Arial 4 pt font, which is a sans-serif font that is easy to read in small font sizes. The sorting data label (32 x 5 mm, Arial 6 pt, two lines) specifies the person who sorted the sample and the taxon. Full vials are topped up with ethanol, leaving as little air as possible to prevent degradation of the specimens, before they are sealed with a polyethylene lid. The vials are then placed in cardboard boxes (designed to hold 49 vials in a 7 x 7 configuration) according to taxonomic fraction. To make individual vials easier to retrieve from the storage boxes, a round self-adhesive label – 16 mm in diameter and made from matte white, chlorine-free bleached cellulose paper – is attached to the top of the lid. The label specifies the trap ID, collecting event ID, trap emptying date, taxon and total number of vials of this taxon in the sorted sample (Fig. 9b).

Sorting procedures for the remaining tiers are similar to those for first tier sorting. One difference is that Chalcidoidea and Braconidae samples are placed into smaller 1.5 ml vials; these are stored in commercially available Sarstedt cardboard boxes with room for 81 (9 x 9) vials each.

As each tier is completed, a data form is filled out specifying the number of tubes of each taxon encountered. The hard copies of these forms are kept in a binder at Station Linné and the data are uploaded through a dedicated client developed by the DINA project (http://dina-project.net) to the Specify collection management system at the Swedish Museum of Natural History. The data are available from the Swedish natural history collection web portal “Naturarv” (http://naturarv.se), as well as from the Global Biodiversity Information Facility (http://gbif.org) and the national Swedish biodiversity data portal (http://bioatlas.se) as a separate dataset (“SMTP Collection Inventory”).

All sorting is conducted according to the standardised tiered system by trained technicians, taxonomists not being allowed to “cherry pick” material from raw SMTP samples. The reason for this is twofold. First, it eliminates unnecessary handling of the material that can damage small, delicate and fragile specimens. Second, there is a risk that removal of select groups may result in the loss of specimens belonging to other groups that are picked out by mistake. Only final fractions from the sorting process are made available to specialists interested in working on SMTP material.

### Training

**Figure 10.**
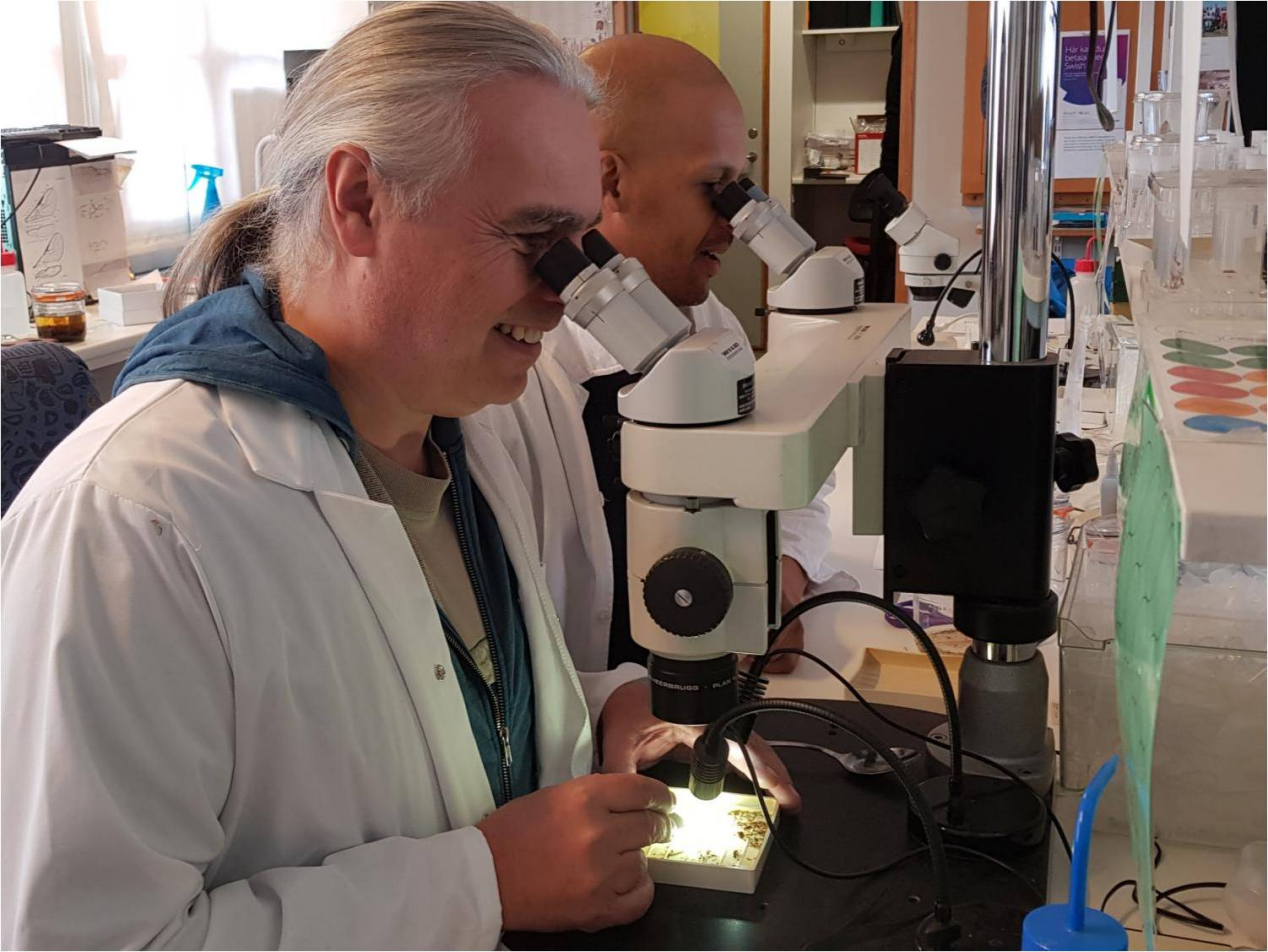
Training session conducted at a double-headed microscope.

The SMTP has trained sixty technicians over the course of the project, with four to ten working in the lab at any one point in time. To ensure high accuracy and efficiency in the sorting process, all technician candidates (whether paid or voluntary) go through a training and testing process. This process includes sorting sessions with real samples conducted alongside veteran technicians using a double-headed microscope (Fig. 10). These joint sorting sessions make it easy for candidates to be shown diagnostic characters and for different handling techniques to be demonstrated. As trainees gain knowledge and confidence, they can begin to take over the sorting from the trainer. This process may take just a couple of hours for an experienced entomologist and up to a week for a student new to insect diversity. In addition to one-on-one training, the SMTP has developed its own identification manuals to aid trainees in acquiring the identification skills necessary to work for the project.

Candidates must pass minimum requirements on identification accuracy before being formally accepted into the project. After initial training, error rates must be under 1%. Skilled candidates may have error rates much lower after the initial training period and, after several months of practice, error rates can be as low as 1 out of 3,000 specimens. Candidates must pass standards for each tier of sorting they wish to undertake, but all sorters need to pass the standard first-tier sorting test. Experienced candidates applying for more specialised SMTP sorting jobs will have their skills tested in a similar fashion.

The SMTP has employed dozens of technicians over the years, both full-time and part-time, sometimes long-term and sometimes seasonally. Many were employed in cooperation with the Swedish Public Employment Service (Arbetsförmedlingen) and the Swedish Social Insurance Agency (Försäkringskassan). Soon after the project began, sorting of the SMTP material at Station Linné attracted the attention of local students and volunteers. A range of interested persons, from high school students to retirees, were interested in contributing to the project while learning new skills and gaining unique insights into Swedish insect diversity. Volunteers are admitted on the same terms as hired staff and have come to play a major role in the sorting tasks over the years.

### Taxonomic Work

Sorted SMTP material is made available to taxonomists around the world on condition that the material is identified to the lowest taxonomic designation possible (species-level identifications are preferred) and that reference material of all species is returned. If new species are described from the material, the holotype and part of the remaining type series must be deposited in the collections of the Swedish Museum of Natural History. The specialist may keep the remainder of the type series and any additional material can be dissected, sequenced or otherwise utilised as desired. Material is delivered in ethanol and sorted to the desired taxonomic fraction. This allows experts to process the material as desired (dissections, mounting, sequencing etc. are all choices left to the specialists).

Most taxonomic processing of SMTP material is accomplished by specialists that have been actively recruited by the project, often on recommendation from other taxonomists. To date, more than 130 experts in 24 countries on four continents have received material from over 170 of the 300 fractions and many of these experts have delivered some quantity of identifications. The ultimate goal is to have experts actively working on all 300 taxonomic fractions, but this is a challenging and perhaps unrealistic goal for several reasons. Many taxonomic professionals work on SMTP material outside of their primary research and skilled amateurs often hold "day jobs" and can commit limited time and resources to identification. No stipends are offered by the SMTP for identifications, but separate grants are funded annually by the STI for taxonomic research on critical groups.

Once an expert is found for a taxon, we begin with a small delivery of material to evaluate both the willingness of the expert to prioritise the processing of SMTP samples and the suitability of the material to the expert’s research needs. In addition to the specimens themselves, experts are sent appropriate data labels (either as digital copies for their own printing or as printouts on archive-quality paper) and an Excel file for returning their identifications. The Excel file contains data on all SMTP trap IDs and collecting event IDs, as well as a list of all known Swedish species of the target group extracted from Dyntaxa (https://www.dyntaxa.se). Typically, the only information that the taxonomists need to provide is the species name, number of specimens, trap ID and collecting event ID. Upon return, the Excel files are checked and ingested using custom tools into the same data flow as the SMTP inventory data.

### Size and Composition

To give those embarking on inventory projects some idea of what they might expect from similar efforts, we analysed the size and taxonomic composition of the total catch. We also looked at the variation in composition over seasons and along a latitudinal gradient (contrasting southern with northern samples). The sorting process does not include counting of the number of specimens in each fraction, as this is time-consuming. Therefore, to estimate the size and composition of the catch, we randomly selected 38 samples and counted the number of specimens in each fraction resulting from first-tier sorting. We also counted the number of specimens in the fractions resulting from second-tier sorting of Diptera (16 samples) and Hymenoptera (34 samples). Data were analysed using R, version 3.6.1, as detailed in the Results section. The data files and R scripts are available from https://github.com/ronquistlab/SMTP.

## Results

### Field Campaign

The use of volunteers as trap managers worked well. Most trap managers followed instructions meticulously and the material was in excellent shape when SMTP staff picked it up. Collected material was often stored under near-ideal conditions in root cellars or non-heated outbuildings or even in refrigerators or freezers. Most traps were maintained continuously for the three-year campaign with the exceptions of a single trap on Öland (Trap 19) that was never emptied (due to site host’s poor health) and another trap (Trap 25) that was operated for just five months before the site host gave up and dismantled the trap. Ten sites (Traps 52-61) along the Vindeln river in Västerbotten province were operated by staff of the Swedish University of Agricultural Sciences for just two seasons, concurrent with another project.

Thankfully, not a single Malaise trap was wilfully sabotaged, despite some of the traps being in high traffic areas. For instance, the trap at Sandhammaren in Skåne (Trap 1005) was placed along the edge of the sand dunes of a public beach that has up to 3,000 visitors per day in the summer. It remained untouched for the 12 months it was deployed there. The trap on the military training ground Marma skjutfält in Älvkarleby (Trap 6) was, on one occasion, gunned down by the military with a star shell fired during a night exercise. This resulted in an upset military officer calling Dave Karlsson and ordering him to “remove the rubbish you have put on our training field”. After learning more about SMTP’s mission and its permission to collect insects on this site, the officer changed his mind and the burnt residues of the old trap were replaced by a new one that survived the rest of the collecting campaign unscathed.

**Figure 11.**
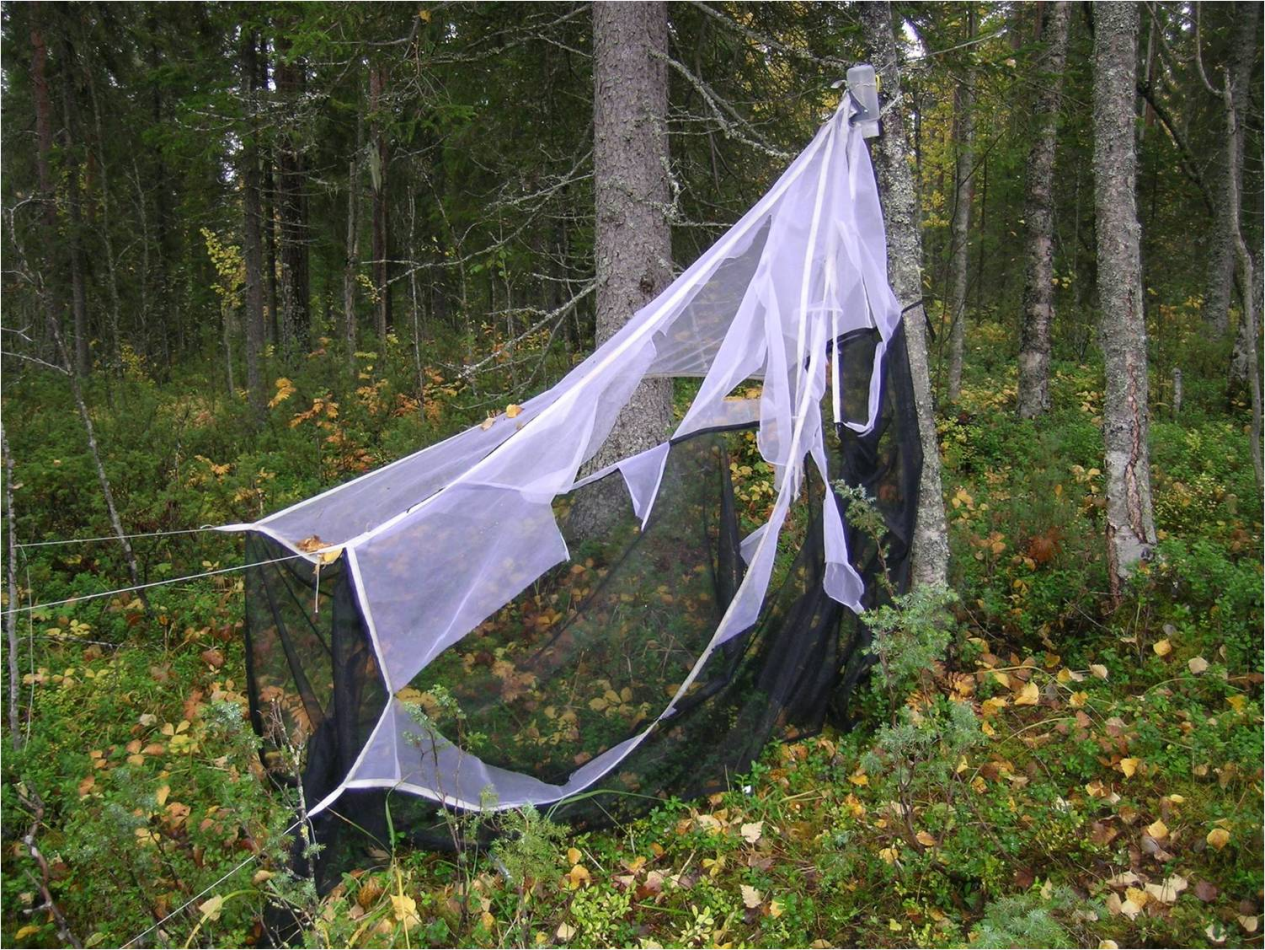
Trap 51, destroyed by a bull moose.

Animals were not as kind to our traps as humans. In September 2004, the trap at Brännbergets Nature Reserve in Västerbotten (Trap 51) was destroyed by a moose bull rubbing his antlers against the trap (Fig. 11). The trap on the mountain Nuolja in Torne Lappmark (Trap 1007) was attacked and eaten (!) by a group of some 20 reindeer, an incident that moved the trap manager to protect the replacement trap with an electric fence (Fig. 1). The trap at Gamla Skogsby on Öland (Trap 22) was initially set up across a roe deer path and was run down by the deer three times in the first year. The problem was partly solved by moving the trap ten metres from the path, though the deer continued to use the trap as a shelter from time to time. Finally, the trap on the Great Alvar of Öland (Trap 20), situated at the border of grazed pastureland, had to be protected by an electric fence to avoid damage from cattle.

### Size of Samples and Total Catch

Trap samples vary considerably in size and composition. Some summer samples have been estimated to contain tens-of-thousands of specimens, while many winter samples contain very few. Traps in the North and in the mountains were covered by snow for several months in the winter, during which time they could not be emptied at all. In contrast, traps in southern Sweden had to be emptied more often than every two weeks during the summer because the collecting jars would otherwise fill up completely with insects. The most extreme case was the trap at Drakamöllan in Skåne (Trap 38), which had to be emptied every four to five days under optimal insect trapping conditions.

The 38 samples, used to estimate the total size and composition of the catch (the "statistics samples"; Table 1) contained a total of 304,839 specimens. The fraction of summer samples (collected in May to August) was not significantly different from that of the remainder of the catch (58% versus 63%, chi-square test, *p* = 0.60). The counted samples were from slightly higher latitudes than the uncounted samples (60.6°N compared to 59.3°N, Welch t-test, *p* = 0.04), but the number of trap days per sample was not significantly different (23.9 versus 28.4, Welch t-test, *p* = 0.09). Thus, the counted samples appeared to be roughly representative of the entire catch.

**Figure 12.**
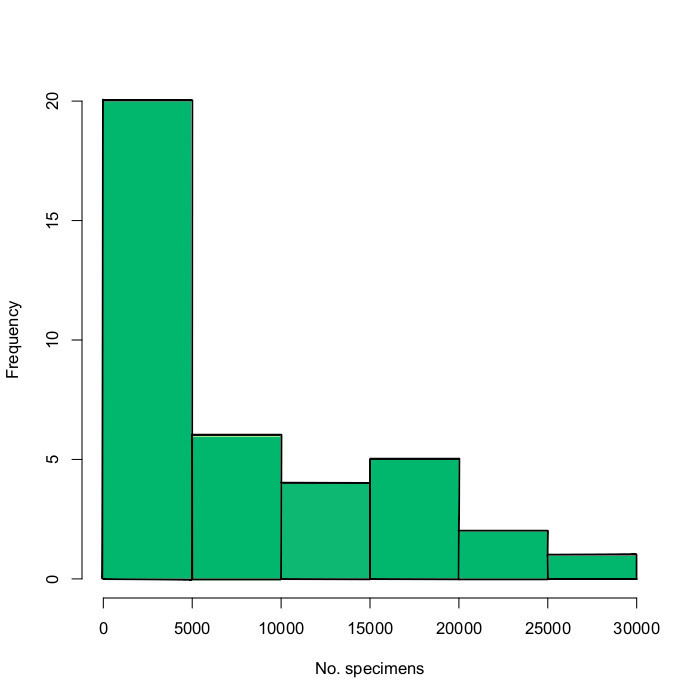
Frequency of total number of specimens per sample.

**Figure 13.**
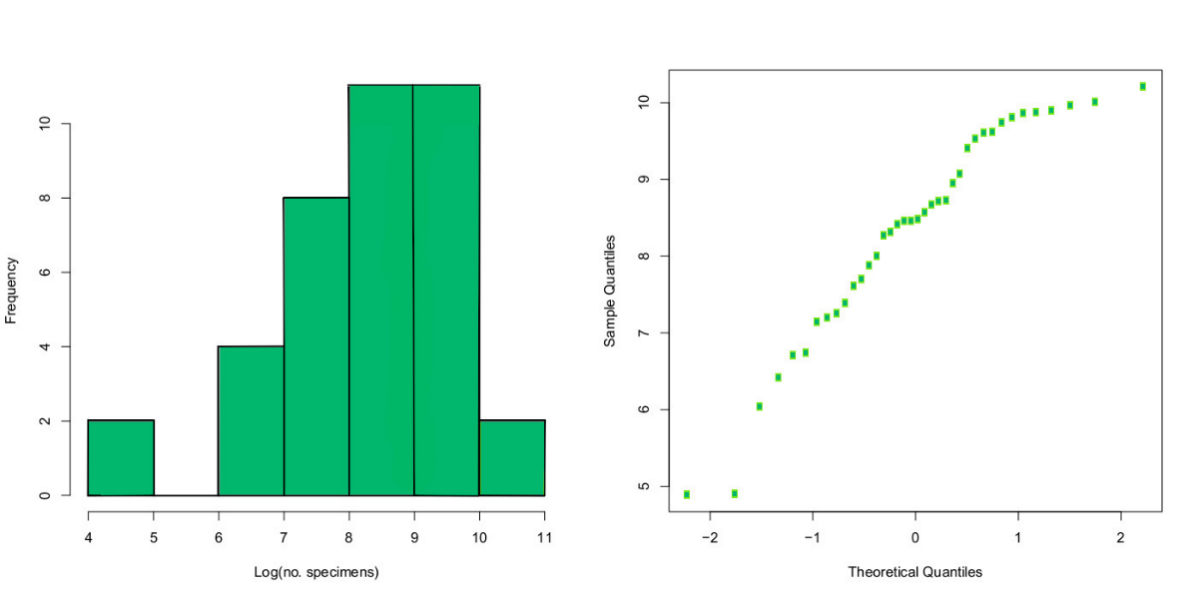
Log transformation of the frequency of total number of specimens per sample (left) and a normal Q-Q plot of the log number of specimens per sample (right), suggesting that the distribution of the specimens per sample may fit a log-normal density.

The total number of specimens in the counted samples varies widely (Fig. 12). The smallest sample contained 132 specimens and the largest 27,206 specimens. The distribution is highly skewed towards small samples. Log transformation suggests that the distribution may fit a log-normal density, which is supported by a normal Q-Q plot of the log number of specimens in the samples (Fig. 13).

**Figure 14.**
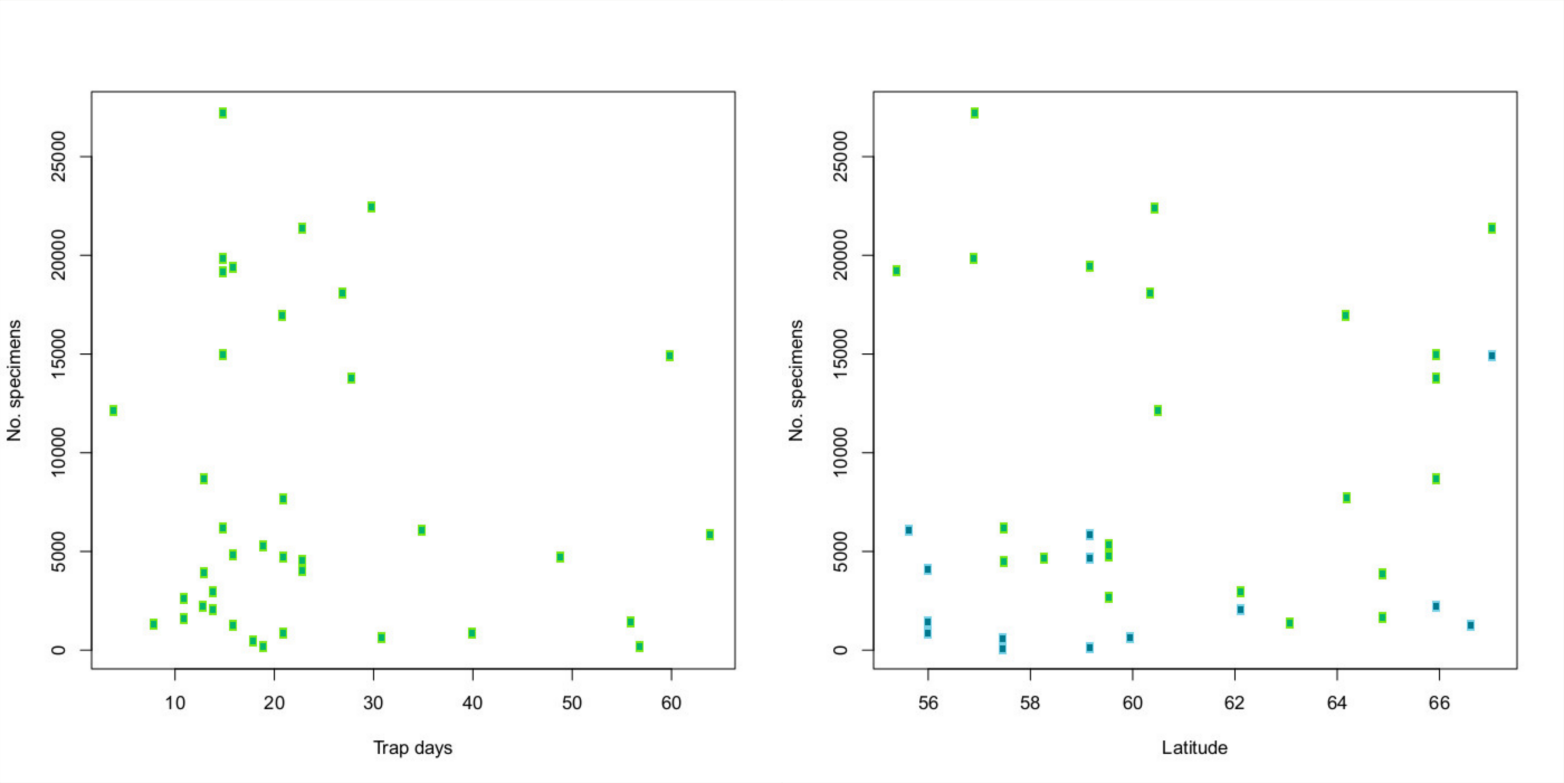
Number of specimens per sample plotted versus trap days (left) and latitude (right). On right, summer samples are displayed in green and non-summer samples in blue.

We used a linear model to examine the influence of trap days, season (summer or non-summer) and latitude on the number of specimens in the sample (Fig. 14). Only season had a significant effect (*p* < 0.002). The same result was obtained if the number of specimens caught per trap day was used as the response variable, with season and latitude as predictors. Using the logarithm of the response variable produced identical results, except that the summer effect was even more significant.

Estimating the total size of the SMTP catch from the counted samples is not trivial because of the huge variance in the number of specimens per sample. The counted samples contain an average of 8,022 specimens. Assuming that this is representative of the entire catch, the total size of the SMTP catch is estimated at 15.4 ± 2.4 million specimens (using the standard error of the mean to represent uncertainty). To obtain a potentially improved estimate, using the fact that the sample sizes appear to fit a log-normal distribution, we first fitted a log-normal distribution to the number of specimens in the statistics samples (using the R function ‘fitdistr’). Then we inferred the total size of the catch from this distribution by repeatedly drawing 1919 samples from it and summarising those. To represent uncertainty about the true values of the log-normal distribution parameters, the log-normal distribution parameters were redrawn for each simulation from a normal distribution centred on the maximum likelihood estimate of that parameter and with the standard deviation set to the standard error of the estimated parameter value. This procedure resulted in an estimate of 21.6 ± 7.0 million specimens. In contrast to the simple extrapolation of the mean number of specimens, this estimate better accommodates the fact that, if we draw from the distribution a large number of times, we are likely to encounter some samples with a very large number of specimens and these will have a large impact on the size of the total catch.

We also tried to estimate the total size of the catch by using data for some abundant taxonomic groups for which a large number of samples had been processed and all specimens identified. The idea was to use the statistics samples to find a model that allowed us to predict the total number of specimens in the processed samples from the number of individuals of the target groups in those samples. This estimate for the processed samples can then be extrapolated to the entire catch. Specifically, we used Phoridae (n = 103 processed samples), Coleoptera (n = 103), Trichoptera (n = 108), Dolichopodidae (n = 390) and Drosophilidae (n = 356) (see Table 2). The processed samples and the statistics samples for these groups were similar in the proportion of the samples that contained the group, the number of specimens of the group, the trap days, the average latitude and the fraction of summer samples (Table 1). The only exceptions were the Phoridae and the Coleoptera: the processed samples had significantly fewer specimens of those taxa (potentially biasing estimates of the total catch downwards), the processed Coleoptera samples represented significantly more trap days than the statistics samples (uncertain effect) and the processed Phoridae samples were from significantly lower latitudes than the statistics samples (uncertain effect).

We fitted both a model with the number of specimens (linear model) and the log of the number of specimens (log-linear model) of the target taxon as predictor of the total size of the sample, in both cases without an intercept, using only the statistics samples that contained the taxon. The log-linear model consistently performed better than the linear model, as indicated by adjusted *R^2^* values (Table 2). Predictions were generated from this model in two steps. First, we predicted the total catch of all groups in the processed samples, using the parameters of the fitted model. Then we extrapolated this to all samples, taking the percentage of samples containing the taxon in question into account. Predictions generated in this way were fairly congruent for the log-linear model, ranging from 12.7 (Coleoptera) to 21.5 (Dolichopodidae) million specimens. The two lowest estimates are for the Coleoptera and Phoridae (12.7 and 15.3 (million?), respectively); both could be on the low side because the processed samples seem to contain unusually small numbers of specimens of those taxa (Table 1); if these groups are removed, estimates range from 17.8 to 21.5 million specimens.

Summing up the different estimates, it seems likely that the total SMTP catch contains around 20 million specimens. However, the uncertainty remains high. For instance, a 95% confidence interval, constructed from the log-normal fitting exercise, would span from 8 to 35 million specimens; naïve extrapolation from the mean of the statistics samples would yield a 95% confidence interval from 11 to 20 million specimens.

### Taxonomic Composition of the Catch

**Figure 15.**
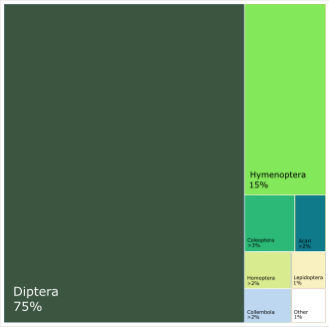
Overall catch composition by percent.

**Figure 16.**
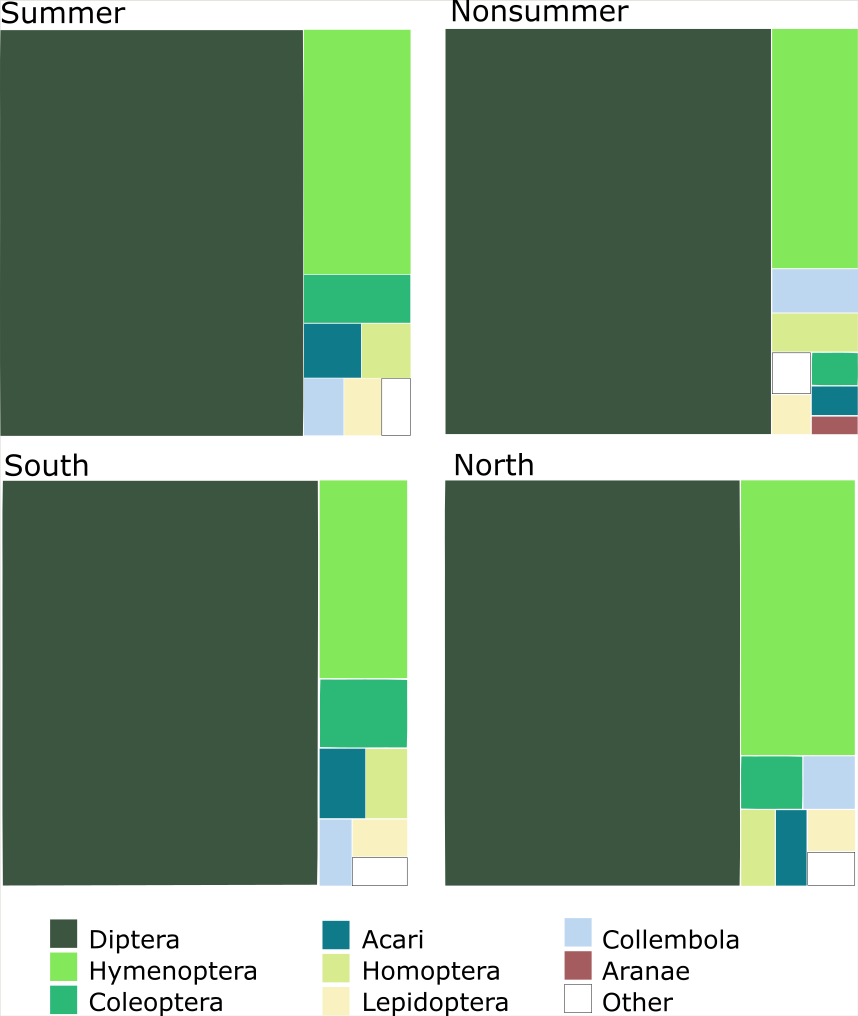
Overall catch composition by season and latitude.

As indicated by the counted samples, the overall catch consists mainly of Diptera (75% of specimens) and Hymenoptera (15%); other insect orders together comprise less than 10% of the total (Fig. 15). The proportions of different groups vary slightly according to latitude and season (Fig. 16). Samples from the north (latitude > 60°N) contain a larger proportion of Hymenoptera than samples from the south (19% versus 11%). Diptera tend to be a smaller percentage of summer samples (collected in May to August), while the reverse is true for Hymenoptera. Collembola are a considerably larger percentage of samples in non-summer than in summer samples (2.3% versus 1.4%).

**Figure 17.**
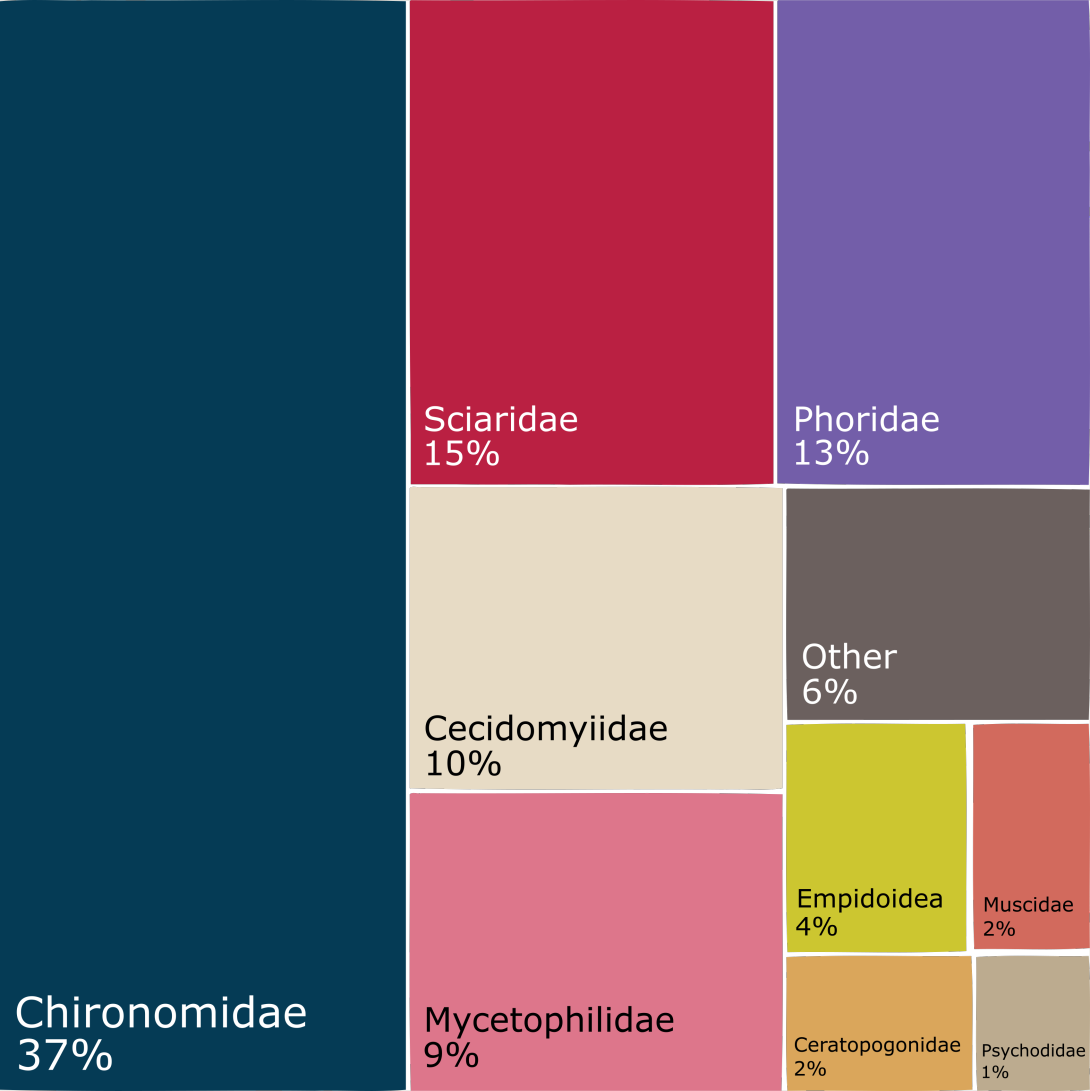
Diptera catch composition.

**Figure 18.**
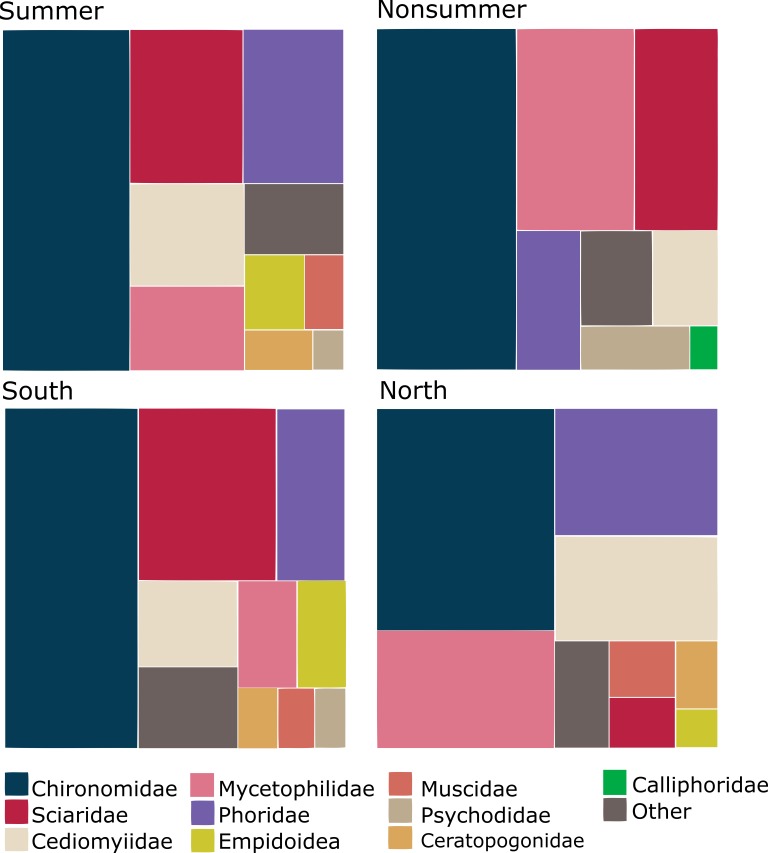
Diptera catch composition by season and latitude.

The dominant Diptera groups are Chironomidae (37% of specimens), Sciaridae (15%), Phoridae (13%), Cecidomyiidae (9.5%) and Mycetophilidae (9.4%) (Fig. 17). The composition varies considerably by season and latitude (Fig. 18). There are groups that comprise a higher percentage of catches in the summer and in the south (Empidoidea) or the reverse (Mycetophilidae). A couple of groups make up a higher percentage of samples in the summer and in the north (Cecidomyiidae and Phoridae), while the Sciaridae are at higher percentages of samples in the summer at all latitudes.

**Figure 19.**
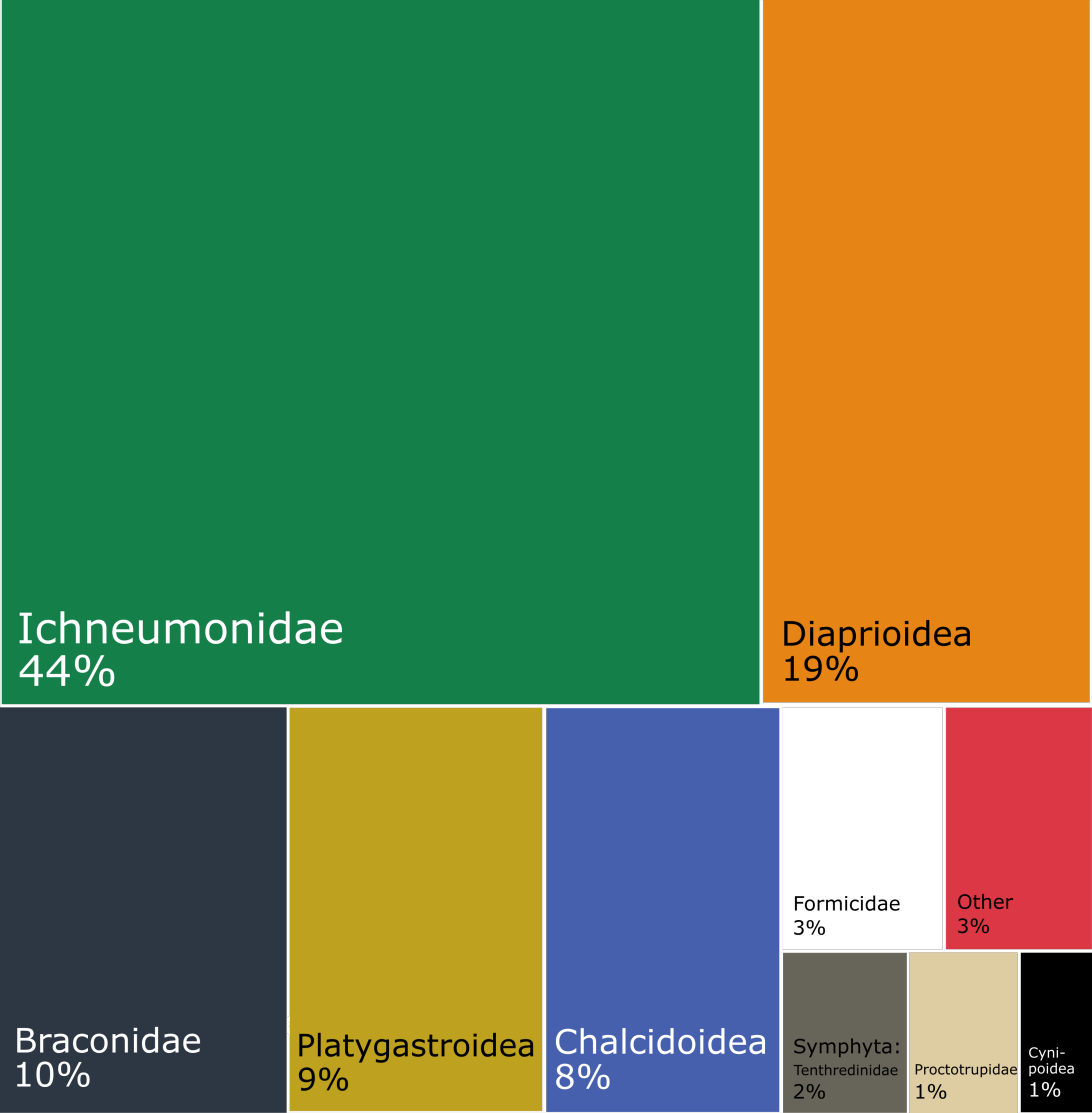
Hymenoptera catch composition.

**Figure 20.**
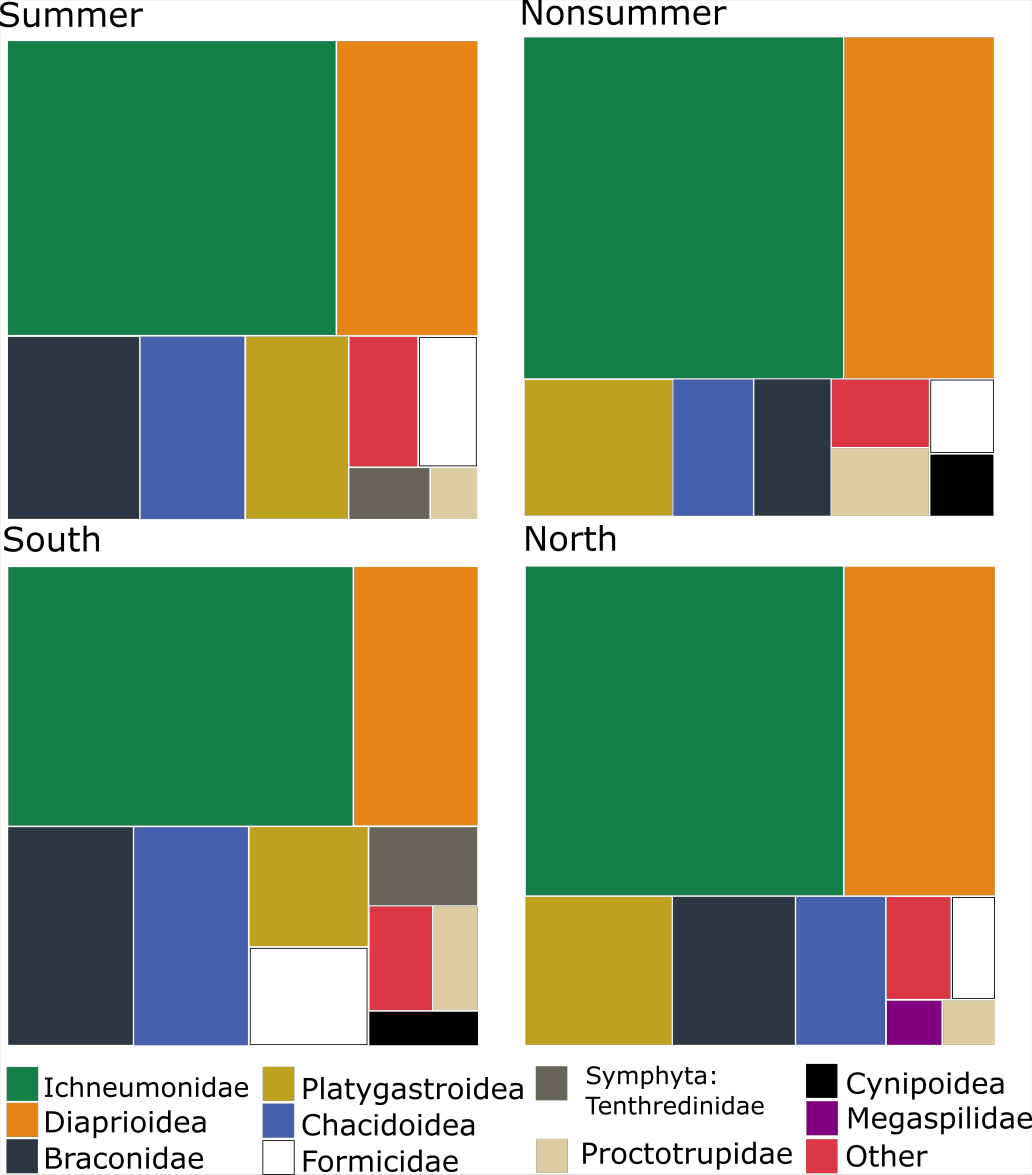
Hymenoptera catch composition by season and latitude.

Within Hymenoptera, the dominant groups in terms of proportions of the total catch are Ichneumonidae (44%), Diaprioidea (19%), Braconidae (9.6%), Platygastroidea (8.5%) and Chalcidoidea (7.9%) (Fig. 19). As in the Diptera, the composition varies according to season and latitude (Fig. 20). Several groups (Ichneumonidae, Diaprioidea, Platygastroidea) make up larger percentages of summer samples in the south, while other groups show the opposite pattern (Braconidae, Chalcidoidea, Formicidae). A couple of groups (Proctotrupidae, Cynipoidea) make up higher percentages of non-summer samples than in summer samples, but there are nevertheless higher percentages of southern samples than northern.

### Person Hours and Project Cost

Contributions of volunteers have been essential to the success of the SMTP. The total person-hours spent on the project are estimated at 138K, with 24K of these (17.3%) performed by volunteer staff. As a percentage of the total *sorting* hours (98K), volunteers contributed nearly one quarter. Site hosts alone contributed an estimated 600 volunteer person-hours to trap maintenance and bottle changing. We have not attempted to compile data on the person-hours contributed by our many collaborating taxonomic experts, as this would be exceedingly difficult to estimate.

The total cost of the project (funding received 2002-2018) is approximately $3.1 million USD, with the majority of funds spent on personnel. Funds of $1.2 million USD have been spent paying sorting staff and $665K USD have been spent on project administration and planning. Costs have been kept low for sorting due, in large part, to shared employment costs with Arbetsförmedlingen and Försäkringskassan for many of the paid technicians. The remaining funds have gone to overheads and project supplies.

To date, the per specimen cost of the SMTP has been $0.16 USD, assuming that the total catch comprises 20 million specimens. If we further assume that each specimen, on average, has passed through sorting in two tiers, then the total number of sorting hours (98K) correspond to a handling time of each specimen of roughly 9 seconds. Data on two staff members show that they sort samples to order (first-tier sorting) at a sustained speed of 270 ± 100 (mean ± standard deviation) specimens per hour (*n* = 5 samples containing a total of 24,372 specimens). This corresponds to an average specimen handling time of 13 seconds. Counting the number of specimens in each of the first-tier fractions was done at a speed of 470 ± 100 specimens per hour (*n* = 6 samples containing a total of 24,985 specimens), corresponding to a specimen handling time of 7.7 seconds.

### Sorting progress and transfer to taxonomic experts

The first tier of sorting is complete for all samples but the additional tiers of sorting for the hyper-diverse orders have only been partly completed. As of this writing (summer 2019), 85% of the Hymenoptera and 70% of the Diptera material have been sorted through the second tier.

Approximately 626,000 specimens have been sent out to experts around the world for identification and 165,000 specimens have been identified to date. Taxonomic work on project material has added 1,303 species to the Swedish fauna. Of these, 87 have been described as new to science and 602 are putative new species that still await formal description (Ronquist et al. 2019). The identified material is focused, to a large extent, on poorly known insect groups. The SMTP maintains a list of all publications using project material and detailed taxonomic data on the project website (http://www.stationlinne.se/sv/forskning/the-swedish-malaise-trap-project-smtp/smtp-publications and http://www.stationlinne.se/en/research/the-swedish-malaise-trap-project-smtp/taxonomic-units-in-the-smtp). Data on the identified specimens are available in separate datasets for each taxonomic group from GBIF (https://gbif.org) and from the Swedish biodiversity data hub, the Bioatlas (https://bioatlas.se).

Several taxa that have previously been considered extremely rare or difficult to collect in Sweden have been encountered in large numbers in the SMTP material. For instance, one of the braconid taxonomists working on SMTP material, Thorkild Munk, had only seen a single specimen of the rare taxon Gnamptodontinae (Braconidae) before being involved in the SMTP inventory. To date, several hundred gnamptodontines have been encountered in the sorted SMTP material. Another striking example concerns the Mymarommatidae (Hymenoptera), which are extraordinarily tiny wasps. Prior to SMTP, only three specimens of *Mymaromma anomalum* (Blood & Kryger, 1922) were known from Sweden. Almost 1,000 specimens of this species plus two specimens of a new species to Sweden (*Mymaromella* sp.) are now available from the SMTP, showing that the taxon is abundant and widespread. A final example is Strepsiptera males, which are quite scarce in most entomological collections, but have been found by the hundreds in the SMTP samples.

Even in well-studied groups, the SMTP material has contributed significantly to our knowledge of the distribution and abundance of the Swedish species. Even though only a tiny fraction of the available material has been processed to date, several new provincial records have already resulted for familiar species of Coleoptera, Diptera, Hemiptera, Hymenoptera, Psocoptera and Thysanoptera.

## Discussion

### Size and composition of the catch

The scale of the SMTP, with an estimated 20 million specimens collected and processed, is perhaps unique amongst inventories; at least, we are unaware of any effort that has processed near this number of specimens morphologically. The processing of entire Malaise trap samples remains quite rare even for much smaller inventory projects. Most Malaise trap samples are processed by having targeted groups removed for study and the bycatch is either shelved (often indefinitely) or even discarded.

As the processing of entire samples remains rare, little information is available in literature on the composition of Malaise trap catches. What data are available confirm the dominance of the order Diptera (75% of SMTP specimens), but are more variable with respect to Hymenoptera (15% of SMTP specimens). A small comparative study in the Neotropical region examined the order-level (excluding Lepidoptera) Malaise trap catch from three samples: two in the same locality in Peru (one using a Malaise trap suspended just above the forest floor) and one in Costa Rica (Brown 2005). In all three samples, Diptera dominated the catch (at 84, 81 and 64%, respectively), followed by Hymenoptera, Coleoptera, Homoptera, Collembola and other orders in smaller percentages (Brown 2005). A study using a single Malaise trap over one year in the Orongorongo Valley in New Zealand caught 45,965 arthropods, 84.2% of which were Diptera, followed distantly by Collembola (4.9%), Hymenoptera (4%), and other orders in smaller percentages (Moeed and Meads 2012). A review by van Achterberg et al. on different types of flight interception traps confirms the efficiency of Malaise traps for capturing Diptera and Hymenoptera, although ratios of these orders and of families within orders, is clearly variable (van Achterberg et al. 2010). Inventories focused on molecular data tend to reveal similar compositional data, although it is often unclear how representative the sequenced specimens are of the entire catch. In two Malaise trap catches from Germany, Diptera represented 70.3% of the individuals analysed (and 51.6% of the BINs detected) (Geiger et al. 2016). Similarly, a large dataset of 939.6K barcoded specimens from across Canada was comprised of 65.4% Diptera, with Hymenoptera coming in second with just 13.4% of successfully sequenced specimens (Hebert et al. 2016). Together, the two orders represented two thirds of the barcode index numbers detected (Hebert et al. 2016). It should be noted, however, that barcoding success was lower for Hymenoptera than for other insect orders in this project. The previously mentioned Global Malaise Trap Program reported their catch to be 55% Diptera, followed by 17% Hymenoptera, when 860K specimens had been sequenced (http://biodiversitygenomics.net/site/wp-content/uploads/2018/02/GMP-Progress-Report-2017.pdf).

Malaise trapping is clearly associated with an inherent bias favouring the capture of some taxa over others. Thus, the clear domination of Malaise trap catches by Diptera may at least partly be due to the fact that Malaise traps are particularly effective in catching many Diptera groups. Similar biases are likely to affect many insect groups, such that the composition of Malaise trap catches is surely different from that of the true insect fauna at the trapping sites. Even the Diptera diversity is only partly sampled well by Malaise traps. An excellent demonstration of this is given by the Zurquí All Diptera Biodiversity Inventory (ZADBI), conducted by 59 taxonomic experts in Costa Rica (Borkent et al. 2018). The ZADBI team used a wide variety of methods to supplement two Malaise traps and the Malaise trap catches together represented only 65% of the total diversity observed (Borkent et al. 2018).

Unfortunately, the ZADBI project did not compile abundance data from processed samples. A comparison for our Diptera composition data was found in the aforementioned Neotropical sampling effort by Brown (Brown 2005). In his family-level analysis of Diptera from four Malaise trap catches, Cecidomyiidae was, by far, the most abundant family at three of the sites (both Peruvian sites and a Bolivian site not included in the previously discussed order-level analysis), while Phoridae were slightly more abundant than Cecidomyiidae at the Costa Rican site (Brown 2005). Other abundant Diptera families were Sciaridae, Ceratopogonidae and Sphaeroceridae, but the numbers varied significantly between sites (full data available on http://phorid.net/phoridae/crisis_index.html). This contrasts with the dominant Diptera groups found in the SMTP: Chironomidae (37%), Sciaridae (15%), Phoridae (13%) and Cecidomyiidae (10%). Similarly, a study on Diptera from seven rainforest sites in Australasia found that three families, Phoridae, Cecidomyiidae and Chironomidae, made up more than half the total catch from three sampling methods (including Malaise trapping) (Kitching et al. 2005). While family composition is apparently quite variable across regions and habitats, it is clear that certain families reliably turn up in abundance.

### Contributions to Sweden's national ATBI and beyond

The SMTP is a product of, and funded by, the STI, Sweden's national ATBI. Therefore, the principal aim of the SMTP is to contribute to the identification of all multicellular life in Sweden (the mission of the STI). The SMTP has been a primary source of study material for taxonomic research on the Swedish insect fauna in recent years. As the STI discoveries of new Swedish taxa and new taxa to science are dominated by insects, the SMTP has contributed in a major way to the overall outcome of STI. Before the start of STI, the Swedish insect fauna was estimated to contain 24,700 species (Gärdenfors et al. 2003). Currently, around 28,000 species are known from the country. Many of the species, new to the country and to science, were first discovered in SMTP material. A substantial fraction of the new species await description and recent estimates, based largely on the SMTP catch, suggest that the true Swedish insect fauna may comprise as many as 33,000 species – an increase of more than 33% compared with the Gärdenfors et al. estimate (Ronquist et al. 2019). Such a dramatic rise in figures is astounding given that Sweden is both a country in Europe, the continent with the best-explored biota and a nation with a long and proud tradition in insect taxonomy.

Many of the SMTP’s collaborating taxonomists come from outside Sweden and are not working primarily on the Swedish fauna. Therefore, SMTP specimens are used as representatives of the Nordic fauna in numerous studies, sometimes even for larger biogeographic regions. This is especially relevant in extremely poorly studied groups for which information on species distributions is scant. In such groups, the STI and the SMTP material may also form the basis of contributions that go far beyond biogeography. For example, in the gall midge family (Diptera: Cecidomyiidae), specimens studied from Sweden provided a major basis for the taxonomic revision of four of the five basal subfamilies, which eventually led to a new classification of the family (Jaschhof and Jaschhof 2009, Jaschhof and Jaschhof 2013).

Importantly, the SMTP and the STI demonstrate to the international community that *we can* do it: *we can* successfully tackle an ATBI on a countrywide scale. Furthermore, they clearly show that the biological diversity of Europe is far from being fully explored; if many species remain to be discovered in Sweden, this must be true for most European countries. These ideas might seem obvious in Sweden itself, where a government-funded, nationwide floral and faunal inventory has been running for so many years. These initiatives must not, however, be taken for granted. Quite simply, comparable projects do not exist in most countries, despite the fact that they have committed to the Convention on Biological Diversity to make inventories of their national floras and faunas. A notable exception is Norway, which launched its own taxonomy initiative in 2009, collaborating closely with the Swedish initiative. Once the political and intellectual atmosphere needed to pursue widespread ATBI projects has spread more widely in Europe and elsewhere, we hope that the SMTP experience can serve as a useful reference for the planning of coming inventories of national insect faunas.

### Station Linné as host institution

It might seem strange that a project like the SMTP is located at a field station in rural Sweden, instead of being run from a university or natural history museum in a big city. However, this choice of location has proven advantageous to the project in many ways. Rural areas lack the many distractions of big cities and simultaneously attract many nature lovers. This makes the station and its efforts natural subjects of public attention and municipal integration. Without the support of the Öland community, neither the station nor the project would likely have prospered during the last decade in the way that they have.

The SMTP has generated a unique and invaluable collection, both in quantitative and qualitative terms, a majority of which is stored at Station Linné. The collection is managed and curated in close collaboration with the Swedish Museum of Natural History in Stockholm (Naturhistoriska riksmuseet, NRM). A selection of research-relevant specimens, including all type specimens of new species, are regularly transferred to and permanently deposited at NRM. Specimens are housed at Station Linné in modest but modern storage facilities; the majority of the material has been stored for several years in darkness at -18°C. The upkeep and overhead costs are low, thanks to the rural location, but the quality standards are comparable to those of a large institution.

With an estimated 20 million sorted insects, the SMTP collection is more than half the size of some of the world’s most impressive natural history collections, such as the NHM London (34 million specimens) or the Smithsonian (35 million specimens). The conditions of the collection are unique: specimens are sorted to taxonomic fractions, but most remain otherwise unprocessed; they are, therefore, not individually curated specimens (as in the aforementioned institution figures) nor unprocessed samples. The majority of specimens reside in ethanol (the exception being Lepidoptera, that are dried), ready for further processing as desired by experts (dissection, sequencing, slide mounting, drying etc.). There is little doubt that the sorting format of the SMTP is a major factor in successfully appealing to taxonomists to work on project material. The SMTP collection provides scientists and students around the world with a rare resource in terms of clean sorted specimens from a plethora of insect taxa, a gold mine for any taxonomist. Station Linné also offers attractive on-site accommodation options and convenient lab facilities for visiting researchers interested in studying the collection.

### Lessons learned

Malaise traps are an economical way to collect large quantities of a wide diversity of insects, but they are not without limitations and drawbacks. A chief limitation is that, even within groups that are generally well represented in Malaise trap samples, there are often taxa that are un- or under-represented. The magnitude of these group-specific sampling biases has become increasingly clear as the SMTP material has been processed and analysed. Additional, complementary collecting methods would undoubtedly have added to the insect diversity sampled in the project, but this would have necessitated a significant reduction in the number of sampling sites given the time, personnel and financial constraints. It is still not clear whether the original approach of using only Malaise traps was the optimal way of inventorying the Swedish insect fauna or whether an approach using more types of traps at fewer sites would have been better. To attempt to capture some of the insect diversity missed by the SMTP, a new inventory, The Swedish Insect Inventory Project (SIIP), was initiated by Station Linné in 2018. This new effort combines the use of Malaise traps with canopy traps, pan traps and interception traps at 37 sites across Sweden and is expected to generate insect material comparable in size to the SMTP material. Comparing the results from these two projects will give some insight into the relative efficiency of the Malaise-trap-only versus the multi-trap approach.

Any project involving mass collecting and processing of specimens may damage or degrade delicate insects to the extent that they become unusable for taxonomic work. For SMTP specifically, the bulk collection of specimens in jars could result in some damage already in the field; for instance, the movement by larger insects when they fall into the jars could damage the remaining specimens. Furthermore, fragile insects always suffer some damage when they are handled and sorted from bulk samples, no matter how refined the techniques or how thorough the training. We tried to minimise these problems in several ways, for instance by disallowing cherry-picking of groups from the bulk samples. Nevertheless, we were not successful enough with some of the most delicate groups, for which targeted collection and expert handling may be needed for satisfactory results. Jaschhof and Jaschhof (Jaschhof and Jaschhof 2009) describe in detail why they prefer to collect and process their own samples of Cecidomyiidae (gall midges) instead of relying on SMTP material. This is based on experience from several years of studying specimens from the SMTP, where up to 50% of cecidomyiid specimens were unsuitable for morphological identification. It is quite possible that alternative strategies could have produced better SMTP material of groups like cecidomyiids. One idea that might be worth trying is to empty the traps more often, which should reduce the damage occurring in the field while insects accumulate in the collecting jars. Furthermore, it is possible that improved initial storage and more careful handling of the samples could help. It may also be worthwhile to experiment with lower concentrations of ethanol or alternative preservative liquids.

In hindsight, more effort should have been devoted initially to the planning and implementation of sample storage routines. Unsorted SMTP samples are now kept in 80% ethanol and sorted samples in 95% ethanol, in both cases at -18°C. However, initially many samples were stored at room temperature and the alcohol concentration was not monitored properly, so the concentration became inadequate for proper conservation in some samples. In addition, in some cases, samples were not collected or stored under ideal conditions during the field campaign, before being brought to the storage facility. Storing the quantity of samples associated with a project like SMTP would probably be challenging for most museums. However, we would strongly recommend ensuring adequate storage facilities before the project starts, instead of adding and improving the facilities during the project, as was the case for SMTP. There is still some uncertainty whether the imperfection in storage routines have affected the sample quality. The molecular work, conducted thus far, has been successful in many cases, but less so in others. Organised trials are needed to determine to what extent the problems that have been experienced are due to imperfect conservation of part of the material or to other factors.

Perhaps the greatest challenge (and hardest lesson) of the SMTP was that of proper project management. Handling such a large project in an organised and efficient manner is a truly monumental task. It involves the logistics of managing and processing thousands of samples, millions of specimens, dozens of employees and volunteers, thousands of data points, hundreds of specimen sheets and data files. Add to this the staff turnover that is likely to occur in the span of 15 years and you have a recipe for chaos. In hindsight, it is obvious that we should have spent more effort on putting proper management routines in place when the project was started, instead of improving those routines as the project developed. Hopefully, this paper provides information that will be helpful in addressing the management challenges in a more orderly fashion in other large-scale insect inventory projects to come in the future.

## Conclusions

The SMTP has proven that large-scale insect inventories are feasible with traditional morphological methods. We argue that, not only are such inventories possible, they are critical. Recent years have seen a number of large-scale molecular inventories initiated. However, the end results of these projects are quite different from those achieved in a morphological inventory like SMTP. In the SMTP pipeline, the material is sorted into fractions suitable for transfer to taxonomic experts. In contrast, the end result of a molecular (barcoding) pipeline is a dataset containing all successfully sequenced specimens categorised by BINs and associated voucher specimens. These BINs are matched to identifications, if they exist, but at this point in time, the legwork to create reliable, comprehensive databases of BINs has not been done. This is clearly shown with a dataset from two German traps that obtained unambiguous species names for just 35% of their BINs (just under 34% of their specimens) (Geiger et al. 2016). Many studies analyse results to whatever level the current BIN databases allow and no further, limiting the usefulness of such efforts (Hebert et al. 2016). Results of molecular projects are, therefore, difficult to compare directly with the SMTP. For example, the Global Malaise Trap Program (GMP; https://biodiversitygenomics.net/projects/gmp/) is a Malaise trap megaproject with 158 sites in 33 countries. Thus far, that project has only processed about 10% (2 million specimens) of the material sorted by the SMTP into taxonomic fractions. However, all the processed specimens are associated with BINs, so it may be more fair to compare the GMP output, not to the 20 million insects sorted, but to the roughly 0.6 million SMTP specimens currently identified or in the process of being identified by taxonomic experts. This comparison, however, is equating BINs to identified species. As discussed above, this is problematic given our current state of knowledge, so comparison of these approaches remains difficult.

Another method used in molecular inventories (e.g. Insect Biome Atlas, https://www.insectbiomeatlas.com/) is the metabarcoding of entire samples using high-throughput sequencing platforms. These inventories aim to lower processing times and costs by sequencing samples en masse, rather than individually sequencing specimens. Metabarcoding generates lists of BINs for a sample, but does not associate specimens with their barcodes. Therefore, any morphological work must essentially be carried out starting with the unsorted sample. While current protocols are often destructive to samples, methods are under development to leave specimens both morphologically and molecularly intact for further study (Marquina et al. 2019). We will undoubtedly see progress on this front in the near future, potentially allowing large-scale inventories to combine metabarcoding, individual sequencing and morphological approaches for optimal efficiency.

The problem of associating BINs to taxonomic and biological information remains a monumental task. Tackling voucher specimens from barcoding projects is one possibility, but this is not often made favourable to taxonomists due to material condition or logistics. We need projects like the SMTP to get quality material into the hands of experts who can identify, describe and communicate the diversity of their groups. Only after this work has been done can sequences in databases be matched with names, morphological details and life history information, recorded by specialists. This, of course, requires that experts barcode the material they work on, something that we consider an imperative next step in the processing of material.

In addition to the processed material, the SMTP has produced a slew of side benefits, many of which are uniquely associated with a morphology-focused inventory project (as opposed to a purely molecular inventory). The project has facilitated the entomological education of numerous students, volunteers and visitors to the station. It has inspired other research projects and inventories and contributed to numerous masters and doctoral theses ("theses and reports" at http://www.stationlinne.se/sv/forskning/the-swedish-malaise-trap-project-smtp/smtp-publications/). The biodiversity research of a nation has fundamentally changed in just 15 years in ways that would not have been possible if the project had been carried out differently. Sweden stands poised, perhaps better than any other country in the world, to fully document its insect fauna in the foreseeable future.

Naturally, it is only with proper funding and a dedicated team that an effort like the SMTP has been possible and for this, we are indebted to the Swedish Taxonomy Initiative and countless contributors to the project in various forms. Luckily, our experiences over the past 14 years have shown that, once a project like SMTP has gained momentum, it reaches a stage of self-fertilisation — a phenomenon important to mention here because it might help future initiatives to dispel initial concerns. The SMTP started with a single employee in 2003 and has since employed dozens of staff members and welcomed scores of eager volunteers, students and collaborators. Station Linné has even welcomed two peripheral taxonomic projects, funded by the Swedish Taxonomy Initiative to the station. These projects, focused on Cecidomyiidae and Phoridae, are tackling two of the most difficult groups of Diptera in close collaboration with the SMTP.

More than a decade has now passed since SMTP's primary collecting effort. The sorting of the original campaign material is coming to a close and the focus will soon shift to the 2018 inventory material. This will provide countrywide data that can be compared with baseline data from the original SMTP. Recent reports of massive insect decline have been met with questions and criticism, in part due to the lack of substantial baseline data (Hallmann et al. 2017, Lister and Garcia 2018, Sánchez-Bayo and Wyckhuys 2019). We hope that our collection efforts may provide solid evidence of the status of the insect fauna of Sweden over the past decade and solidify the SMTP as a pivotal inventory in understanding our insect fauna.

## Figures and Tables

**Table 1.** Descriptive statistics for the taxa and the samples used to estimate the total catch. The samples used to infer the size of the total catch are referred to as "statistics samples". The other samples for which the specimens of the taxon have been counted and identified are referred to as "determined samples". The proportion of samples containing the taxon are given for the entire catch (estimated) and for the statistics samples. To check whether the statistics samples appeared to be representative of the entire catch, we tested for significant differences in the specimen count between the determined samples and the statistics samples using the Wilcoxon test.

	Number of samples identified or containing the taxon		Proportion of samples with taxon		Specimen count (mean ± standard deviation)		Difference in specimen count (significance)
Taxon	Determined samples	Statistics samples	Entire catch	Statistics samples	Determined samples	Statistics samples	
Phoridae	103	37	0.97	0.97	428±445	1024±1293	*
Coleoptera	103	36	0.94	0.95	49±68	236±466	***
Trichoptera	108	19	0.62	0.50	33±217	12±33	ns
Dolichopodidae	390	11	0.77	0.69	111±607	87±122	ns
Drosophilidae	356	12	0.77	0.75	21±50	19±17	ns

**Table 2.** Estimates of total catch using various taxa with both linear and log-linear regression models.

	Linear model			Log-linear model		
Taxon	Adjusted R^2^	Significance	Prediction	Adjusted R^2^	Significance	Prediction
Phoridae	0.70	***	4.7	0.69	***	15.3
Coleoptera	0.54	***	1.5	0.77	***	12.7
Trichoptera	0.16	*	11.7	0.49	***	19.6
Dolichopodidae	0.47	**	16.7	0.82	***	21.5
Drosophilidae	0.31	*	14.9	0.58	**	17.8
